# Control Targets in Plant-Pathogenic Bacteria: From Growth-Essential Processes to Anti-Virulence Strategies and Candidate Targets in *Candidatus* Liberibacter Asiaticus

**DOI:** 10.3390/plants15142150

**Published:** 2026-07-12

**Authors:** Jinyin Zeng, Chenyu Huang, Yuxun Yu, Xiaobing Song, Meirong Xu, Xiaoling Deng, Bo Wang, Zheng Zheng

**Affiliations:** 1 Citrus Huanglongbing Research Laboratory, South China Agricultural University, Guangzhou 510642, China; 2 Institute of Plant Protection, Guangdong Academy of Agricultural Sciences Key Laboratory of Prevention and Control on Fruitsand Vegetables in South China Ministry of Agriculture and Rural Affairs, Guangdong Provincial Key Laboratory of High Technology for PlantProtection, Guangzhou 510640, China

**Keywords:** plant-pathogenic bacteria, control target, anti-virulence, quorum sensing, biofilm, type III secretion system, copper resistance, *Candidatus* Liberibacter asiaticus

## Abstract

Plant-pathogenic bacteria threaten crop productivity and quality, yet chemical options remain limited compared with those for fungal and oomycete diseases. Current management relies mainly on copper bactericides, limited antibiotics, induced-resistance agents, biocontrol and resistant cultivars. However, copper and streptomycin resistance, efflux-mediated multidrug tolerance and rapid pathogen adaptation have weakened these strategies. Target-oriented research provides a framework for exploring agricultural antibacterials, anti-virulence agents, anti-colonization strategies, resistance sensitizers and host-resistance interventions, but many of these approaches remain conceptual, model-system, greenhouse or medical-bacteriology-derived rather than proven field solutions. This review classifies bacterial control targets into two interconnected groups: growth-essential targets, including peptidoglycan biosynthesis, membrane/envelope systems, nucleic-acid processes, protein synthesis, metabolism, nutrient transport and cell division; and anti-virulence/anti-adaptation targets, including secretion systems, quorum sensing, biofilms, motility, adhesion, cell-wall-degrading enzymes, tolerance systems, oxidative-stress responses and host susceptibility factors. Using “*Candidatus* Liberibacter asiaticus” (CLas) as a case study, genome annotation and infection-stage transcript-abundance data prioritized Sec-dependent secretion, outer-membrane/surface proteins, Bam assembly, nutrient transporters, Clp proteostasis, redox adaptation and core cellular processes as candidate target classes. Envelope-associated, secretion/anti-virulence, nutrient-acquisition and stress-sensitization modules may represent potential directions for downstream validation, but CLas candidates remain hypothesis-generating priorities requiring validation for essentiality, conservation, druggability, delivery feasibility, crop safety and field performance.

## 1. Introduction

Plant-pathogenic bacteria inhabit diverse ecological niches, including leaf surfaces, the rhizosphere, vascular tissues, fruits and storage environments, and cause many economically important crop diseases [1]. Representative groups include foliar pathogens such as *Xanthomonas* and *Pseudomonas syringae* [2,3], the vascular wilt pathogen *Ralstonia solanacearum* [4], the fire blight pathogen *Erwinia amylovora* [5], and soft-rot pathogens such as *Dickeya* and *Pectobacterium* [6,7,8,9]. Other important taxa include *Agrobacterium*, which causes crown gall disease [10]; *Xylella fastidiosa*, which is associated with xylem-limited diseases [11,12]; and *Candidatus* Liberibacter asiaticus (CLas), the principal bacterial species associated with citrus Huanglongbing (HLB) [13,14,15,16]. These pathogens differ markedly in their ecological niche, transmission route and pathogenic mechanism. For example, foliar pathogens commonly cause leaf spots, cankers or necrosis, vascular pathogens induce systemic wilt or xylem/phloem-associated diseases, and soft-rot bacteria secrete plant cell-wall-degrading enzymes that lead to tissue maceration [2,3,4,6,11].

Current management of bacterial plant diseases relies on integrated measures, including clean planting material, quarantine, reduction in primary inoculum, pruning and sanitation, vector suppression, protectant treatments, biological control and resistant cultivars [17,18,19,20]. Copper compounds remain important protectants for many bacterial leaf spot and canker diseases because of their broad spectrum, low cost and long history of use [21,22,23,24]. Streptomycin, oxytetracycline and related antibiotics have also been used in fire blight and a limited number of high-value crop systems [17,19,20,25,26]. Nevertheless, the diversity of molecular targets and product pipelines for agricultural antibacterials remains far smaller than that for fungicides [18,20]. Moreover, ultraviolet radiation, rainfall wash-off, plant-surface waxes, vascular barriers and microbial-community interactions can reduce the accessibility, persistence and field performance of antibacterial agents [27,28,29,30,31].

During the past two decades, copper resistance, streptomycin resistance and multidrug tolerance have been reported in multiple plant-pathogenic bacterial populations [20,21,23,26,32]. The environmental release of agricultural antibiotics may also contribute to the dissemination of resistance genes, prompting stricter risk assessment and management [17,19,20,25,26]. In this context, control-target research should not be restricted to direct bactericidal discovery. It increasingly includes anti-virulence, anti-colonization, anti-biofilm, anti-transmission, sensitization and host susceptibility-factor interventions [33,34,35,36,37]. This shift does not diminish the importance of bactericidal targets; rather, it highlights the need to select target combinations according to the pathogen niche, transmission biology, delivery feasibility and translational scenario [38,39,40,41,42].

Based on current advances in molecular plant bacteriology, this review adopts a two-category framework for organizing bacterial control targets. The first category comprises growth-essential bactericidal or bacteriostatic targets that affect pathogen proliferation and survival, including the bacterial cell wall, outer membrane, nucleic-acid metabolism, ribosomes, metabolism and cell division [38,42,43,44,45]. The second category comprises anti-virulence, anti-colonization and anti-adaptation targets that interfere with completion of the infection cycle. Secretion systems and effectors are central components of this category [46,47,48,49,50]. Additional intervention points include quorum-sensing and biofilm-associated processes [51,52,53,54], resistance and sensitization systems [32,38], and host susceptibility factors [55,56,57,58].

Finally, CLas is used as a case study not as a separate target-discovery project, but as an application of the framework developed in the preceding sections. The general target classes reviewed above provide mechanistic categories, whereas the prioritization criteria summarized in Table 1 provide the decision logic for evaluating whether a candidate is biologically relevant, potentially accessible, experimentally tractable and translationally feasible. This framework is particularly useful for CLas because its phloem-limited lifestyle, reduced genome, low abundance and lack of routine artificial culture make conventional antibacterial screening difficult. Therefore, the CLas section applies the same conceptual criteria to integrate genome annotation, infection-stage transcript abundance, predicted localization, mechanistic target class and validation feasibility into a hypothesis-generating candidate list.

Additionally, although the infection niche and delivery route are central to agricultural translation, this review does not aim to provide a comprehensive formulation- or delivery-technology matrix. Instead, delivery feasibility, cellular accessibility, field stability and safety are incorporated as prioritization criteria to ensure that molecularly attractive targets are interpreted cautiously in relation to plant-surface, vascular, phloem or vector-associated constraints.

## 2. Conceptual Framework for Control-Target Classification

**Figure 1:**
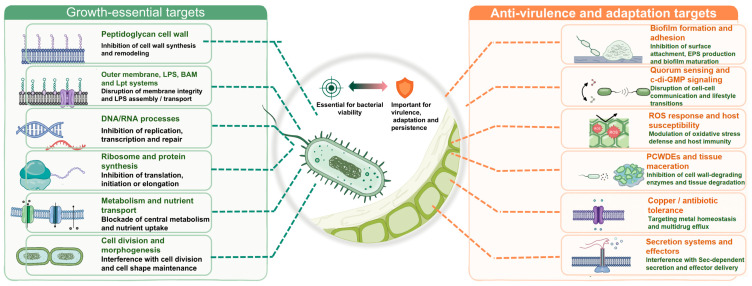
Conceptual framework for control targets in plant-pathogenic bacteria. Growth-essential targets provide direct biochemical endpoints, whereas anti-virulence and anti-adaptation targets reduce pathogenicity, colonization, transmission or stress tolerance. Both categories should be evaluated in relation to delivery barriers, resistance risk, selectivity and compatibility with integrated disease management. Targets with overlapping functions, such as Sec secretion, Bam assembly, Clp proteostasis and redox-adaptation systems, are classified according to their primary biological role, whereas secondary roles in virulence, stress adaptation, accessibility or sensitization are considered during prioritization.

Control targets in plant-pathogenic bacteria can be classified according to their cellular location, biological process and intended application (Figure 1). From a spatial perspective, they include targets associated with the outer membrane, cytoplasmic membrane, periplasm, cell wall, cytoplasm, extracellular matrix and host–cell interface [38,39,40,41,42]. Functionally, major target groups include cell-wall synthesis, membrane assembly, nucleic-acid metabolism, protein synthesis, metabolism and nutrient transport [38,42,43,44,45]. Secretion systems and effectors represent a distinct virulence-associated module [46,47,48,49,50], whereas signaling regulators, biofilm formation, motility, adhesion, stress adaptation and host-interaction processes provide additional intervention points [33,37,51,54,59]. In terms of the application purpose, these targets can be further considered as bactericidal or bacteriostatic targets, anti-virulence targets, anti-colonization targets, sensitization targets, delivery-associated targets or host-side resistance targets [33,34,35,36,37].

Growth-essential targets usually have defined biochemical functions and measurable in vitro screening endpoints, making them suitable for enzyme-activity assays, structure-guided drug design and high-throughput screening [38,39,40,41,42]. Their major advantages are direct antibacterial activity and relatively mature validation systems [39,40,41]. However, their translational value in plant-pathogenic systems may be limited by strong resistance selection and uncertain chemical accessibility in complex infection niches, particularly on leaf surfaces and within xylem or phloem tissues [27,28,29,30,31].

By contrast, anti-virulence and anti-adaptation targets do not necessarily inhibit bacterial growth in vitro, but instead reduce colonization, tissue damage, vector transmission or suppression of host immunity [33,34,35,36,37]. These strategies may impose weaker direct selection for growth-based resistance under some conditions, but this should not be interpreted as a universal or resistance-proof property. In agricultural ecosystems, repeated treatments, large pathogen population sizes, heterogeneous exposure on plant surfaces or within tissues, and strong fitness advantages during infection can still select for mutants with reduced sensitivity to anti-virulence or anti-adaptation interventions. Resistance or loss of efficacy may arise through target modification, regulatory bypass, functional redundancy, compensatory mutations, increased efflux, biofilm-mediated tolerance or shifts in pathogen population structure. Therefore, evolutionary durability should be evaluated using serial-passage assays, in planta competition experiments, fitness-cost analysis, mixed-population assays and field resistance monitoring. Anti-virulence and anti-adaptation approaches are likely to be most useful when combined with copper bactericides, biological control agents, induced-resistance agents, resistant cultivars or other integrated disease-management measures, rather than used as stand-alone resistance-proof strategies.

The distinction between these categories should be viewed as functional rather than absolute. Outer-membrane assembly systems maintain envelope integrity while also influencing surface antigens, phage receptors and compound permeability [38,42]. Sec systems mediate general secretion and membrane-protein localization, but also support the export of multiple secreted effectors [60,61,62,63,64]. Similarly, Clp, Lon, DnaK and thioredoxin systems maintain protein quality control and redox homeostasis, while also contributing to stress adaptation inside host tissues [39,40,41,42,65,66]. Therefore, target evaluation should integrate essentiality, infection-stage expression, conservation, accessibility, selectivity, delivery feasibility and resistance risk rather than relying on a single criterion.

The two-category framework should therefore be interpreted as a functional organizing scheme rather than a mutually exclusive classification. For targets with overlapping roles, classification was based on the primary biological process most directly associated with the target, whereas secondary roles were incorporated during prioritization. For example, the Sec system was assigned primarily to protein secretion and export, but its contribution to membrane-protein localization and effector deployment was also considered. The Bam complex was treated primarily as an envelope-assembly module, while its effects on surface exposure, outer-membrane permeability and delivery accessibility were considered as secondary prioritization features. Clp proteases and chaperones were classified mainly as protein-quality-control and stress-adaptation targets, but their possible roles in virulence regulation and in planta fitness were also considered. Similarly, redox systems were treated mainly as stress-adaptation and sensitization targets, although they may also contribute to survival under host-derived oxidative stress, copper exposure and other antibacterial treatments.

Such functional overlap affects target prioritization in two opposite ways. On the one hand, multifunctional targets may receive higher priority when they combine mechanistic importance, infection-stage expression, accessibility, conservation and relevance to virulence or stress adaptation. On the other hand, multifunctionality can increase interpretation and translation risks because inhibition may produce pleiotropic effects on bacterial growth, stress tolerance, host interaction and non-target microbial communities. Therefore, overlapping targets were not prioritized simply because they belonged to multiple categories. Rather, their priority depended on whether multiple lines of evidence converged to support biological relevance, validation feasibility and translational potential.

## 3. Growth-Essential Targets and Translational Constraints

Growth-essential targets constitute a foundational category in antibacterial target discovery because they are directly linked to bacterial viability, proliferation and cellular homeostasis. In plant-pathogenic bacteria, their prioritization should also consider target accessibility and infection-niche relevance. This section therefore reviews growth-essential targets as interconnected functional modules, including cell-envelope, membrane and peptidoglycan systems, nucleic-acid processing, protein synthesis and cell division, and metabolism, nutrient acquisition and transport dependence.

### 3.1. Cell-Envelope, Membrane and Peptidoglycan Systems

Most economically important plant-pathogenic bacteria are Gram-negative, and their outer membrane represents both the first barrier to antibacterial entry and a key interface for host recognition, environmental adaptation and surface colonization [2,3,4,38,42]. Lipopolysaccharide (LPS) and lipid A maintain outer-membrane stability and can function as microbe-associated molecular patterns recognized by plants [2,67,68]. Accordingly, LpxC, LpxD, MsbA, LptA–G, LptD/E, BamA/BamD/BamE, Lol systems and outer-membrane porins constitute important components of the Gram-negative envelope target space [38,42]. Outer-membrane assembly and LPS-transport systems may serve as direct antibacterial or sensitization targets [38,42], whereas surface-exposed proteins can provide recognition sites for antibodies, peptides, phages or nanocarriers [30,52,54,69,70]. Modulating membrane permeability may also improve the entry of copper compounds, plant-derived chemicals and other low-toxicity molecules [21,23,24,32,38]. In vascular or uncultured pathogens, such as *Xylella fastidiosa* and CLas, outer-membrane and surface-associated proteins may further serve as diagnostic markers, delivery-recognition sites and anti-colonization targets [11,12,15,16,71]. Where possible, the following discussion prioritizes evidence from phytopathogenic bacteria, including *Xanthomonas*, *Pseudomonas syringae*, *Ralstonia*, *Erwinia*, *Pectobacterium*, *Dickeya*, *Clavibacter*, *Xylella* and CLas. Clinical or model-bacterial examples are included only when they provide mechanistic guidance for target biology, druggability or validation strategy, and should not be interpreted as direct evidence for agricultural disease-control efficacy.

Envelope and LPS systems are active areas of target discovery in Gram-negative bacteria, but the evidence level of specific inhibitors differs markedly between medical bacteriology and plant-pathogenic systems. In plant-pathogenic bacteria, the outer membrane, LPS transport, envelope assembly and membrane permeability are biologically relevant because they influence antibacterial entry, surface recognition, stress tolerance, colonization and interaction with host or environmental factors. However, most well-characterized inhibitors of these pathways have been developed and validated primarily in clinically relevant Gram-negative bacteria rather than in phytopathogenic bacteria. LpxC inhibitors, including L-161,240, CHIR-090, ACHN-975 and LPC-233, have generated systematic structure–activity data in clinical or model Gram-negative species and inhibit an early deacetylation step in lipid A biosynthesis [72]. LptD/E and LptA–G mediate LPS transport across the envelope, and murepavadin/POL7080 provides a representative example of an antibiotic targeting outer-membrane biogenesis through LptD in *Pseudomonas aeruginosa* [73]. BamA and the BAM complex assemble β-barrel outer-membrane proteins, and darobactin, dynobactin and related macrocyclic peptide-like compounds bind BamA at the lateral gate or substrate-recognition region, thereby blocking outer-membrane protein insertion [74]. Non-bactericidal polymyxin derivatives, such as SPR741 and NAB739, can also function as outer-membrane permeabilizers and enhance compound entry into Gram-negative cells [75]. These compounds should therefore be interpreted as conceptually transferable mechanistic references rather than as validated agricultural antibacterials. For agricultural translation, however, outer-membrane permeabilizers and membrane-active compounds should be evaluated with particular caution. Although such agents may enhance antibacterial entry, their broad effects on bacterial membranes may cause phytotoxicity, residues, disturbance of the beneficial phyllosphere, rhizosphere or endophytic microbiota, and regulatory concerns. Therefore, these compounds are currently better regarded as mechanistic tools or sensitization leads unless crop safety, selectivity, degradation behavior, microbiome compatibility and field efficacy are demonstrated in plant disease systems.

Although most of these agents have not yet been translated into plant disease control, their mechanisms provide a useful framework for envelope-targeted strategies against *Xanthomonas*, *Pseudomonas*, *Ralstonia* and CLas. In parallel with these small-molecule mechanisms, several biotechnology-based approaches have advanced for the management of bacterial plant diseases caused by these pathogens. Microbial biocontrol agents and bacteriophage-based formulations have been developed or commercialized for selected diseases caused by *Xanthomonas* spp. and *Pseudomonas syringae*, particularly in vegetable and fruit production systems [76,77]. However, their field performance is usually preventive and context-dependent, and efficacy can be influenced by formulation stability, environmental persistence, pathogen population structure and compatibility with integrated disease management [76,77]. For *Ralstonia solanacearum*, phage therapy, rhizosphere biocontrol, resistant rootstocks and microbiome-oriented approaches have shown promising results, but broad and stable large-scale application remains limited because of the pathogen’s soilborne lifestyle, broad host range, strain diversity and long-term survival in soil [78]. For citrus canker, host-side genome editing has provided a representative biotechnological route; editing TAL effector-binding elements in the *CsLOB1* promoter generated citrus lines with enhanced resistance to *X. citri* subsp. *citri*, although this strategy remains primarily at the experimental and breeding-validation stage rather than routine commercial deployment [79]. For CLas and HLB, emerging strategies include antimicrobial peptides, antisense or RNA-targeting molecules, nanocarrier-assisted delivery, host immune modulation, vector-stage intervention and genome-informed target prioritization. A stable antimicrobial peptide from *Microcitrus australasica* was reported to reduce CLas titer and symptoms in controlled greenhouse assays and to move systemically in citrus tissues, but further field validation and delivery optimization are still required before large-scale use [80]. By contrast, delivery-oriented oxytetracycline trunk injection has been evaluated in HLB-affected citrus and was widely adopted in Florida after registration of injectable OTC formulations, although this practice is not a target-specific biotechnology and its long-term efficacy, durability and resistance-management implications remain under evaluation [81,82]. Therefore, current large-scale applications are mainly represented by registered biocontrol/phage products for selected bacterial diseases and antibiotic trunk injection for HLB management, whereas most target-based, genome-guided or CLas-directed strategies still require further validation of efficacy, delivery, durability, regulatory acceptance and ecological safety.

The bacterial cell wall is essential for maintaining cell shape, resisting osmotic stress and completing cell division [43,76]. Peptidoglycan biosynthesis involves cytoplasmic MurA–MurF reactions, membrane-associated MraY and MurG reactions, and periplasmic transglycosylation and transpeptidation [43,83]. Penicillin-binding proteins, FtsI/PBP3, RodA/PBP2, MraY, MurG, MurA and peptidoglycan hydrolases are therefore candidate targets [43,44,45,83,84]. The translation of classical cell-wall targets to plant pathology should therefore be interpreted cautiously. Although MurA, MraY, PBPs, FtsI/PBP3 and β-lactams are well-established antibacterial targets in medical and model-bacterial systems, direct evidence for their practical use in controlling phytopathogenic bacteria remains limited. In major plant-pathogenic genera such as *Xanthomonas*, *Pseudomonas*, *Ralstonia*, *Erwinia*, *Pectobacterium* and *Clavibacter*, cell-wall and division-related processes are biologically important, but most available discussions still rely on general bacterial cell-wall biology, genome-based target inference or mechanistic extrapolation rather than plant-pathogen-specific disease-control validation. Although many cell-wall targets were first characterized in medical bacteriology, their mechanisms provide useful methodological guidance for developing bacteriostatic agents against plant-pathogenic bacteria [38,39,40,41,42]. In Gram-negative plant pathogens, however, peptidoglycan is located between the inner and outer membranes, so compound entry is strongly influenced by outer-membrane permeability and efflux pumps [38,39,40,41,42]. Cell-wall target studies in *Xanthomonas*, *Pseudomonas*, *Ralstonia*, *Erwinia* and *Pectobacterium* should therefore be combined with outer-membrane permeabilization, efflux-pump inhibition or delivery-carrier design [2,3,4,6,42]. In Gram-positive plant pathogens, such as *Clavibacter*, the greater exposure of cell-wall structures may make peptidoglycan-biosynthesis and remodeling enzymes more accessible [1,43,83].

The inhibitor landscape for cell-wall biosynthesis is relatively mature. Fosfomycin, the classical MurA inhibitor, mimics phosphoenolpyruvate and covalently modifies an active-site cysteine, thereby blocking the UDP-*N*-acetylglucosamine enolpyruvyl transfer reaction [85]. MraY inhibitors include nucleoside natural products and derivatives, such as tunicamycin, liposidomycin, mureidomycin, muraymycin, caprazamycin and capuramycin, which interfere with lipid I formation; however, selectivity, cell entry and stability in plant tissues remain major limitations [86]. PBP and FtsI/PBP3 inhibitors are represented by β-lactams, which have strong and well-defined mechanisms, but their activity in Gram-negative plant pathogens is often limited by the outer-membrane barrier, β-lactamases and efflux pumps [87]. Therefore, these inhibitors are most useful as mechanistic references or as candidates for evaluation with permeabilizers or β-lactamase inhibitors. Overall, small-molecule studies targeting the cell wall in plant-pathogenic bacteria remain less systematic than those in medical bacteriology. Future studies should establish integrated MurA/MraY/PBP enzymatic, cell-morphology and infection-assay platforms for representative pathogens, such as *Xanthomonas*, *Ralstonia* and *Pectobacterium*.

### 3.2. Nucleic-Acid Processing, Protein Synthesis and Cell Division

DNA gyrase, topoisomerase IV, RNA polymerase, DNA polymerases, DNA ligase, DnaA, DnaB, DnaG, RecA and SOS-response factors constitute the principal target set for bacterial nucleic-acid processes [38,39,40,41,42]. Quinolones and rifamycins demonstrated the druggability of DNA topoisomerases and RNA polymerase [39,40,41,42], but agricultural applications must avoid cross-selection with clinically important antibiotics [17,19,20,25,26]. Therefore, GyrB ATPase domains, non-classical RNA polymerase binding sites, DNA ligase and RecA/SOS inhibitors may represent more suitable low-cross-resistance target options [38,39,40,41,42]. RecA and the SOS response are particularly relevant in plant-pathogenic bacteria because ultraviolet radiation, copper ions, reactive oxygen species and antibacterial agents on plant surfaces can induce DNA damage and repair responses [23,24,27,29,32]. If these responses increase mutation rates, they may facilitate resistance development and adaptive evolution [19,26,37]. The RecA/LexA axis should therefore be viewed as an exploratory anti-adaptation or sensitization strategy rather than an established agricultural target. Although RecA/SOS inhibition may theoretically reduce stress-induced mutagenesis and adaptive diversification, disruption of DNA repair could also have unpredictable effects on bacterial persistence, cell death, compensatory evolution and resistance selection under plant and field conditions [33,37,38,40,42]. Its practical value therefore requires careful validation in plant-pathogenic bacteria and infection-relevant systems.

DNA topoisomerase inhibitors include quinolones, oxolinic acid, fluoroquinolones, coumarins targeting the GyrB ATPase domain and newer non-quinolone topoisomerase inhibitors [88]. Oxolinic acid and some quinolone derivatives have shown antibacterial activity in seed-treatment or bacterial-disease studies, but environmental persistence and clinical cross-resistance risks limit their agricultural deployment [88]. Rifamycins are classical RNA polymerase inhibitors that bind the RpoB channel and block early transcription elongation [89]. Other RNA polymerase inhibitors, such as sorangicin, streptolydigin, myxopyronin and corallopyronin, act at sites distinct from the rifamycin-binding pocket and may theoretically bypass some *rpoB*-mediated resistance. For plant-pathogenic bacteria, however, this direction remains largely at the concept-validation or model-bacterium stage and should be evaluated in combination with copper compounds, ROS-enhancing agents or DNA-damaging treatments.

The 30S and 50S ribosomal subunits, EF-Tu, EF-G, aminoacyl-tRNA synthetases, tRNA-modification enzymes, signal peptidase, SecA/SecYEG and tmRNA/SmpB systems jointly maintain protein synthesis and translational quality control [38,39,40,41,42]. Streptomycin targets the 30S ribosome and has been used against fire blight and other bacterial plant diseases [17,19,26,90]. However, the emergence of *rpsL* mutations and resistance genes such as *strA/strB* has limited its sustainable use [19,20,25,26]. Accordingly, new protein-synthesis targets in plant-pathogenic bacteria should prioritize sites or mechanisms with lower cross-resistance risks to clinical antibiotics [20,38,40,42]. Protein-synthesis inhibitors are among the few antibacterial classes with a history of agricultural use. Streptomycin interferes with decoding on the 30S ribosomal subunit, oxytetracycline prevents aminoacyl-tRNA entry into the A site, and kasugamycin acts on the 30S subunit and affects translation initiation. These agents have been used or studied in fire blight, bacterial spot and some rice bacterial disease systems [17,19,26,83]. Aminoacyl-tRNA synthetase inhibitors, such as mupirocin, tavaborole and additional aaRS leads, demonstrate the druggability of this enzyme class, although systematic evaluation in plant pathogens remains limited [83].

Protein-secretion-related targets have both growth-essential and virulence-associated functions. Sec and Tat pathways localize membrane proteins and export periplasmic or extracellular proteins [38,39,40,41,42], while signal peptidase processes secretory precursors [39,40,41,42]. Specific extracellular enzymes or effectors of plant pathogens depend on these pathways for maturation and transport [7,8,60,91,92]. For CLas, which lacks a typical T3SS and instead relies on Sec-dependent secreted proteins for host interactions, the Sec pathway links core cellular processes with anti-virulence intervention [60,61,62,63,64]. SecA inhibitors, signal-peptidase inhibitors and arylomycin-like compounds can block pre-secretory processing or protein translocation and therefore have both antibacterial and anti-virulence potential [93,94]. The ClpP system can be dysregulated by acyldepsipeptide antibiotics, causing uncontrolled proteolysis and bacterial death [95]. In plant pathogens, the links between ClpP/ClpX, T3SS expression, stress adaptation and virulence make this target class worthy of infection-system validation.

Bacterial cell division depends on FtsZ polymerization into a Z ring and coordinated recruitment of FtsA, ZipA, FtsQ, FtsI/PBP3, FtsW and MinCDE proteins to complete septation and peptidoglycan synthesis [44,45,83,84]. MreB, RodA and PBP2 contribute to rod-shaped morphology [44,83]. Because FtsZ is highly conserved and has no directly equivalent essential counterpart in eukaryotic cells, it has long been considered a promising antibacterial target [45,84]. In plant-pathogenic bacteria, FtsZ, FtsI, FtsH and morphology-maintenance proteins may serve as basic bacteriostatic targets, although compound permeability, crop safety and field stability remain key barriers [38,39,40,41,42]. FtsZ inhibitors, including PC190723, TXA707/TXA709, benzamide derivatives and natural-product derivatives such as berberine and sanguinarine, generally interfere with FtsZ polymerization, GTP hydrolysis or Z-ring dynamics [96]. The MreB inhibitor A22 binds directly to MreB, lowers its polymerization capacity and induces abnormal rod-cell morphology [97]. In rod-shaped plant pathogens, such as *Xanthomonas*, *Ralstonia* and *Pectobacterium*, FtsZ/MreB inhibitors can be used for target validation and morphological screening.

Therefore, the agricultural translation of nucleic-acid, transcriptional, translational and cell-division targets requires strict consideration of cross-resistance risks. Clinically important classes, especially fluoroquinolones and rifamycins, should not be considered for routine field use against plant bacterial diseases because they share targets and resistance mechanisms with antibiotics used in human and veterinary medicine. Accordingly, classical quinolones, rifamycins and related benchmark topoisomerase or RNA polymerase inhibitors are discussed here mainly as mechanistic tools or target-validation probes, not as field-deployable agricultural antibacterials. Future exploration should focus only on low-cross-resistance chemotypes or non-classical binding mechanisms with acceptable crop safety, environmental behavior and regulatory feasibility.

### 3.3. Metabolism, Nutrient Acquisition and Transport Dependence

Central metabolism, the respiratory chain, fatty-acid synthesis, folate synthesis, NAD biosynthesis, menaquinone biosynthesis, amino-acid biosynthesis and nutrient transport are fundamental to bacterial survival [38,39,40,41,42]. However, the ecological niches occupied by plant-pathogenic bacteria are highly heterogeneous. Leaf surfaces are nutrient-poor and environmentally fluctuating [27,28,29,30,54], the rhizosphere contains plant exudates and microbial interaction signals [27,28,29], and xylem and phloem tissues impose distinct nutritional and physical constraints [11,15,16,71]. Therefore, essentiality under in vitro culture conditions does not necessarily correspond to essentiality during infection, and metabolic target evaluation should consider infection-stage expression, tissue localization and niche-specific nutrient availability [31,66].

Nutrient transport systems are particularly important in genome-reduced and host-dependent pathogens. CLas and *Xylella* have limited biosynthetic capacities and often depend on host or insect-vector environments for amino acids, organic acids, nucleotides, metal ions and carbon sources [11,15,16,71,98]. ABC transporters, dicarboxylate transporters, phosphate transporters, metal transporters and outer-membrane porins may therefore serve either as nutrient-deprivation targets or as potential entry routes for delivery strategies [38,42,98,99,100]. Metabolic-target inhibitors include dihydropteroate synthase and dihydrofolate reductase inhibitors, type II fatty-acid synthesis inhibitors, NAD-biosynthesis inhibitors, menaquinone biosynthesis inhibitors and MEP isoprenoid-pathway inhibitors [38,39,40,41,42]. Among these, type II fatty-acid synthesis represents a chemically tractable antibacterial target class, although selectivity against plants and environmental microbiota must be carefully assessed [38,39,40,41,42].

Transporter inhibitors remain less developed than many enzyme-targeting strategies because substrate specificity is often uncertain, transporter redundancy is common and infection-niche validation is technically challenging. Nevertheless, in host-dependent and genome-reduced pathogens, metabolic and transporter targets are attractive because they may reveal niche-specific vulnerabilities that are not apparent in standard culture media. Their validation, however, requires infection-relevant assays rather than reliance on in vitro growth inhibition alone. Accordingly, metabolic and transporter targets should be prioritized only when pathway annotation, infection-stage expression and ecological nutrient dependence converge; otherwise, they may remain plausible but non-actionable candidates.

## 4. Anti-Virulence, Anti-Colonization and Anti-Adaptation Targets

Anti-virulence, anti-colonization and anti-adaptation strategies expand control-target research beyond direct growth suppression by focusing on infection, persistence, transmission and stress tolerance. These targets are most valuable when they can be integrated with protectants, induced-resistance agents, biological control, resistant cultivars and delivery systems. However, reduced growth inhibition should not be equated with resistance-proof activity, field efficacy or ecological neutrality. This section therefore considers secretion systems, effectors and host susceptibility axes, quorum sensing, biofilms and colonization systems, and sensitization or stress-adaptation targets as complementary modules for integrated bacterial disease management.

### 4.1. Secretion Systems, Effectors and Host Susceptibility Axes

The type III secretion system (T3SS) is a major virulence apparatus in *Xanthomonas*, *Pseudomonas syringae*, *Ralstonia solanacearum* and some *Erwinia*/*Pantoea* species, which transfer effectors into plant cells through a needle-like apparatus [46,47,48]. These effectors suppress PAMP-triggered immunity, interfere with hormone signaling, alter vesicle trafficking and reprogram host transcription, but can also trigger effector-triggered immunity in plants carrying corresponding resistance proteins [47,49,50,101,102]. T3SS-related targets included Hrp/Hrc structural proteins, HrpG/HrpX, HrpL, Hpa accessory proteins, T3SS ATPases and the effectors themselves [46,47,48,49,50]. By inhibiting T3SS expression, assembly or secretion without substantially suppressing in vitro growth, such compounds may reduce virulence while imposing weaker selection pressure and may complement copper compounds, induced-resistance agents and biological control [33,34,35,36,37].

Representative T3SS-inhibiting compounds have been reported in several plant-pathogenic bacterial systems. Salicylidene acylhydrazides, acylhydrazones, phenoxyacetamides and related compounds established an early proof of concept for T3SS-targeted inhibition in animal and plant pathogens, with some compounds also suppressing T3SS- and amylovoran-biosynthesis-related gene expression in *Erwinia amylovora* [103]. In *X. oryzae* pv. *oryzae* (Xoo), natural phenolic acids and derivatives, such as CZ-1, CZ-4 and CZ-9, inhibited *hpa1* promoter activity, reduced *hrpG/hrpX* and *hrp*-cluster expression, and decreased rice bacterial blight symptoms [104]. Ortho-coumaric acid induced transcriptional responses consistent with T3SS inhibition, further supporting the anti-virulence potential of plant-derived phenolics [105]. In addition, 1,3-thiazolidine-2-thione and 1,3,4-thiadiazole derivatives inhibited *hpa1* promoter activity, hypersensitive response induction and *hrp* gene expression in the *Xoo*–rice system, and showed protective activity comparable to, or better than, traditional agents in controlled assays [106]. Together, these studies indicate that T3SS inhibitors have progressed from a proof of concept toward plant-pathogen infection assays, although their direct molecular targets, resistance risks and field stability remain to be clarified [107].

TAL effectors are a major class of transcription activator-like type III effectors in *Xanthomonas* that enter the plant nucleus, recognize defined promoter sequences and induce host susceptibility genes [108,109,110,111]. In *Xoo*, TAL effectors activated rice *SWEET* sugar-transporter genes and thereby increased host sugar availability to the pathogen [55,56,112,113]. In *X. citri* pv. *citri* (*Xcc*), PthA4-like TAL effectors induced *CsLOB1* and promoted citrus canker lesion formation [57,58]. The TAL effector–susceptibility gene axis therefore provided both pathogen-side and host-side intervention opportunities [55,56,57]. Direct chemical targeting of the TAL–DNA interface was difficult since this interface was broad, sequence-specific and lacked obvious small-molecule pockets [108,109,110,111]. More mature strategies included host-side promoter editing and fine-tuned regulation of susceptibility genes, such as editing TAL effector-binding elements in rice *SWEET* promoters or modifying the citrus *CsLOB1* promoter to reduce induction by PthA4-like effectors [55,56,57,58]. Host susceptibility factors, such as SWEET genes and the *CsLOB1* promoter, are discussed here as complementary host-directed interventions rather than bacterial targets in the strict sense, because they reduce pathogen exploitation of host processes rather than directly inhibiting bacterial growth or virulence machinery. At the chemical-intervention level, T3SS/Hrp regulatory inhibitors or transcriptional-network inhibitors may indirectly reduce TAL expression or translocation, but these strategies should be distinguished from gene-edited resistance [55,56,57]. Future translation of this target axis is likely to rely on molecular breeding, promoter editing and combined anti-virulence strategies rather than broad-spectrum bactericides.

The type II secretion system (T2SS) exported extracellular enzymes and was closely associated with tissue degradation in soft-rot pathogens and some *Xanthomonas* species [7,8,9,91,92]. *Pectobacterium* and *Dickeya* secreted plant cell-wall-degrading enzymes, including pectate lyases, polygalacturonases, cellulases and proteases, which degraded the middle lamella and cell wall and thereby caused tissue maceration, soft rot and leakage [6,7,8,9]. Accordingly, these secreted enzymes, together with T2SS structural proteins (i.e., GspD and GspE) and regulatory factors (i.e., KdgR), represent key anti-virulence targets for soft-rot diseases [7,8,9,91,92]. Compared with T3SS and quorum-sensing inhibitors, T2SS and plant cell-wall-degrading enzyme inhibitors remain less developed. Pectate lyases, polygalacturonases and cellulases can be targeted by plant polygalacturonase-inhibiting proteins, metal or calcium modulation, substrate analogs and enzyme inhibitors, but most studies remain at the enzymology or resistance-breeding level [7,8,9,91,92]. T2SS ATPases, the outer-membrane secretin GspD and pseudopilus assembly factors have drug-target potential, yet mature small-molecule inhibitors remain scarce.

The type IV secretion system (T4SS) of *Agrobacterium* mediates T-DNA and effector transfer and was essential for crown gall formation [10,114,115]. The VirA/VirG two-component system sensed plant phenolic signals and induces *vir* gene expression, whereas VirB/VirD4 formed the transport machinery [114,115]. Interfering with VirA/VirG signaling or VirB assembly might therefore provide anti-*Agrobacterium* strategies and offer a model for understanding pathogen-to-host gene transfer. Acetosyringone analogs, signal-competition molecules and VirB-assembly inhibitors remained conceptual directions for this pathosystem [114,115]. More broadly, small-molecule studies targeting secretion-system regulation, adhesion or other virulence-regulatory pathways supported the feasibility of anti-virulence intervention [33,34,35,36]. However, target-specific and plant-validated inhibitors of the *Agrobacterium* VirA/VirG–VirB/VirD4 system for crown gall control remain limited.

Many bacterial pathogens manipulate host susceptibility factors through effectors or metabolic interference to acquire nutrients, suppress immunity or alter tissue development [49,94,108,109,111]. Rice *SWEET* sugar-transporter genes were well-characterized susceptibility factors induced by *Xanthomonas* TAL effectors [55,56,112,113], while the citrus *CsLOB1* promoter was similarly exploited by PthA4-like effectors during citrus canker development [57,58]. Hormone signaling nodes, stomatal regulators, cell-wall-remodeling enzymes and sugar-efflux pathways may also be considered host-side targets [50,94,95]. These targets are usually not addressed by direct bactericides; instead, molecular breeding, genome editing, induced resistance and precise regulatory strategies can reduce pathogen exploitation of host processes [55,56,57,58]. Host-side approaches may provide durable resistance and reduce direct pressure on environmental microbial communities, but their effects on plant growth, yield, quality and abiotic-stress responses must be carefully evaluated [56,57,58]. Future strategies should emphasize precise promoter editing, tissue-specific regulation and multilocus small-effect combinations rather than simple knockout of genes with essential physiological roles.

Host-side interventions include induced-resistance agents, plant immune modulators, genome-edited materials and disease-control peptides. Acibenzolar-S-methyl (ASM/BTH), a salicylic-acid-pathway inducer, has shown potential to reduce bacterial spot, bacterial speck and bacterial wilt development in tomato systems [116]. β-Aminobutyric acid, chitosan, alginate oligosaccharides, oligogalacturonides, harpin proteins and microbial elicitors can activate or prime basal immunity or systemic acquired resistance, although efficacy depends on the cultivar, treatment timing and environment [117]. Recent studies on the CLas–citrus–psyllid interaction and the MYC2/PUB21–APP3-14 module further suggested that stabilizing key immune transcription factors and disrupting pathogen–vector mutualism may provide indirect routes for HLB control [118,119]. Compared with classical bactericidal targets, secretion-system-, effector- and host-side interventions are relatively advanced in plant-pathogenic bacteria because virulence-reporter assays can be integrated with plant infection models. However, induced-resistance and host-side interventions should be interpreted cautiously because their field performance is often unstable. Defense activation may involve phytotoxicity, growth or yield penalties, metabolic costs, cultivar-dependent responses and strong effects of treatment timing, disease pressure and environmental conditions. Therefore, these agents are most appropriate as optimized components of integrated disease management rather than as stand-alone or universally effective control measures.

### 4.2. Quorum Sensing, Biofilms, Extracellular Matrices and Colonization

Quorum sensing (QS) is a central regulatory layer that coordinates population-level behaviors, including virulence, colonization, biofilm formation and stress adaptation, in plant-pathogenic bacteria [51,120,121,122]. Major QS and related signaling modules include AHL–LuxI/LuxR systems in *Pectobacterium*, *Dickeya*, *Pantoea* and some *Pseudomonas* species [51,59,120,121,122]; DSF–Rpf signaling in *Xanthomonas* and *Xylella*, which regulates biofilm formation, motility and host or vector adaptation [68,70]; and the Phc system in *Ralstonia*, which used 3-OH PAME or related fatty-acid methyl ester signals to control exopolysaccharide production, motility and bacterial wilt virulence [4,123]. AI-2 and c-di-GMP also contribute to intercellular communication, collective behavior and biofilm–motility transitions across diverse bacterial pathogens. QS interference can be achieved by blocking signal synthesis, antagonizing signal receptors, enzymatically degrading signaling molecules, disrupting downstream regulatory cascades or modulating second-messenger levels [51,59,120,122,124]. However, the translational maturity of QS- and biofilm-targeting strategies remains uneven. Many QS inhibitors, c-di-GMP modulators and biofilm-dispersal agents remain supported mainly by reporter assays, in vitro phenotypes or model-bacterial systems, whereas only selected quorum-quenching enzymes, QS-targeting compounds and biofilm-related interventions have been evaluated in plant infection or greenhouse assays. Field-level efficacy and commercial applicability remain limited for most QS- and biofilm-targeting approaches.

Several classes of QS-targeting agents have been explored, including signal-synthesis inhibitors, receptor antagonists, signal-degrading enzymes and downstream regulatory modulators. Halogenated furanones, coumarins, cinnamaldehyde, curcumin, quercetin, resveratrol and other plant-derived phenolics had been reported to interfere with AHL-mediated QS and QS-associated biofilm phenotypes, although their direct application in plant-pathogenic bacteria was often constrained by the effective dose, chemical stability and non-specific biological effects [59,120,121,122,124]. For agricultural use, these compounds also require evaluation of the formulation stability, effective field dose, production cost, crop safety, environmental degradation and non-target effects on beneficial microbiota. Therefore, many QS-interfering small molecules should currently be viewed as mechanistic leads rather than field-ready disease-control agents unless their agronomic performance and safety are demonstrated in plant disease systems. Enzymatic quorum quenching provides a more specific strategy as illustrated by the AHL lactonase AiiA, which degrades AHL signals and attenuates the virulence of *Erwinia*/*Pectobacterium*-type soft-rot pathogens [125]. More target-directed small-molecule progress had been reported for the *Ralstonia* Phc system, in which PQI-1 and optimized analogs PQI-2–PQI-5 inhibited biofilm formation, exopolysaccharide production and ralfuranone production in strain OE1-1, while also reducing tomato wilt symptoms [126]. By contrast, DSF–Rpf systems in *Xanthomonas* and *Xylella* remained largely at the mechanistic or lead-screening stage, with proposed intervention strategies including fatty-acid analogues, inhibition of RpfF-mediated DSF synthesis, disruption of RpfC/RpfG signaling and modulation of c-di-GMP levels [127].

Biofilms are structured bacterial communities embedded in extracellular matrices composed of exopolysaccharides, proteins, lipids and extracellular DNA. In plant-pathogenic bacteria, biofilm formation promotes long-term persistence on leaf surfaces, in the rhizosphere, in vascular tissues and at wound sites, while enhancing tolerance to copper compounds, antibiotics, desiccation, ultraviolet radiation and plant immune responses [30,52,53,54]. Biofilm-associated traits may also influence pathogen acquisition and transmission by insect vectors [70,128,129]. Their biological importance has been illustrated by several major pathosystems. For example, *Xylella fastidiosa* relied on adhesins, biofilm formation and type IV pili for xylem colonization [69,70], whereas *Ralstonia* EPS I contributed to vascular occlusion and wilting [4,116]. In addition, xanthan gum promoted surface colonization and stress tolerance in *Xanthomonas* [2,68], and amylovoran was a major virulence determinant of fire blight caused by *Erwinia amylovora* [5].

Accordingly, anti-biofilm and anti-colonization targets include matrix-biosynthesis systems, matrix-degrading enzymes, extracellular DNA, adhesins, pili, c-di-GMP metabolic enzymes and surface proteins [52,53,54,69]. Representative intervention strategies include enzymatic matrix disruption, signaling interference, anti-adhesion approaches and delivery-enhancing systems. For example, Dispersin B and other polysaccharide hydrolases can degrade specific polysaccharide matrices [130]. Cellulases, pectinase modulators and alginate lyase may further weaken selected matrix components. Other model biofilm-dispersal or biofilm-inhibition agents included N-acetylcysteine, low-dose nitric oxide donors, cis-2-decenoic acid and c-di-GMP-pathway modulators [130]. However, these anti-biofilm approaches differ substantially in their evidence base. Matrix disruption, adhesion interference and biofilm regulation are relevant to several phytopathogenic bacteria, but many specific agents, including Dispersin B, N-acetylcysteine, nitric oxide donors, cis-2-decenoic acid and broad c-di-GMP modulators, have been characterized mainly in medical, model-bacterial or in vitro biofilm systems. Their transfer to plant disease control therefore requires validation of delivery to the infection niche, environmental stability, crop safety, microbiome effects and disease-control efficacy.

### 4.3. Sensitization, Stress Adaptation and Integrated Disease Management

Long-term reliance on copper compounds and antibiotics, such as streptomycin, has selected for diverse resistance and tolerance mechanisms in plant-pathogenic bacteria [26,32]. Copper resistance was commonly associated with metal-transport and detoxification systems, including P-type ATPases, CopABCD, CueO, CusCFBA, Pco systems, metal-responsive regulators, and mobile genetic elements such as plasmids or integrative conjugative elements [21,32]. Streptomycin resistance frequently involved *rpsL* mutations and aminoglycoside-modifying enzymes, such as StrA/StrB and AadA [17,19,20]. In addition, multidrug efflux pumps, including AcrAB–TolC and Mex systems, can reduce susceptibility to multiple antibacterial compounds and contribute to broad-spectrum tolerance [26,38,42].

Sensitization targets are designed not necessarily to kill pathogens directly, but to restore or enhance the activity of existing control agents, reduce application rates and delay resistance spread [33,34,35,36,37]. Potential strategies included copper sensitization, efflux-pump inhibition, resistance-enzyme inhibition, interference with mobile-element-mediated resistance dissemination and suppression of DNA-damage or SOS-mediated adaptive responses [26,32,38]. This approach was particularly relevant to agricultural disease management, because copper compounds and other protectants are unlikely to be fully replaced in the short term [18,32]. However, sensitization strategies require cautious field-oriented evaluation. Copper sensitizers, efflux-pump inhibitors and resistance-enzyme inhibitors may enhance antibacterial activity, but they may also increase phytotoxicity, alter copper mobility in soil, affect beneficial phyllosphere or rhizosphere microbiota, persist in the environment or face regulatory restrictions. Therefore, their practical value should be judged by whether they can reduce input levels and improve integrated disease management without increasing residue, ecological or resistance-selection risks.

Several mechanistic routes can be considered for resistance reversal and sensitization. Copper-resistance systems may be indirectly weakened through metal-transport inhibition, chelation, outer-membrane permeabilization or oxidative-stress synergy, although phytotoxicity and increased soil-metal mobility must be avoided [21,23,32]. Efflux-pump inhibitors were widely used as mechanistic tools in Gram-negative bacteria and can increase sensitivity to diverse antibacterial agents, although field application was limited by toxicity, instability and broad effects on non-target microbes [26,38,42]. Resistance-enzyme inhibition and interference with resistance-gene dissemination may also help restore antibacterial activity or slow the spread of resistance determinants in plant-pathogenic bacteria [17,19,25]. For agricultural applications, these sensitization strategies are best developed in combination with low-dose copper, plant-derived compounds, biological-control metabolites or phage-based treatments to build low-residue and lower-selection-pressure disease-management systems.

Stress-adaptation systems provide a second sensitization axis. During infection and field exposure, plant-pathogenic bacteria encounter reactive oxygen species, metal ions, antimicrobial peptides, pH shifts, osmotic stress and nutrient limitation [27,28,29,30,32]. Regulatory and protective systems, such as OxyR, SoxR/SoxS, KatG/KatE, AhpC/AhpF, thioredoxin, glutaredoxin, ClpP/ClpX/ClpA, Lon, DnaK, GroEL and HtpG, help maintain redox balance, protein folding and cellular homeostasis under these stresses [38,42,65]. Inhibiting these systems may sensitize pathogens to induced plant defenses, ROS-enhancing treatments or copper compounds, thereby reducing their ability to maintain fitness within host tissues [32,33,37].

Oxidative-stress and protein-quality-control targets are therefore best viewed as adaptation-weakening targets rather than classical bactericidal targets. Thioredoxin reductase inhibitors, such as auranofin, perturb bacterial thiol-redox homeostasis and may synergize with host-derived ROS or copper ions [131]. Peroxiredoxins and BCP proteins, together with other bacterial antioxidant systems, such as AhpC and KatG/KatE, were potential ROS-sensitization targets, although selectivity against plant antioxidant enzymes was a critical concern [131,132,133]. Protein-quality-control systems can also be targeted, as illustrated by ADEP-mediated dysregulation of ClpP-dependent proteolysis [95]. However, stress-adaptation targets require careful selectivity assessment. Thioredoxin, Clp proteases, chaperones, ROS-defense proteins and protein-quality-control systems are often conserved and may overlap functionally with plant stress-response pathways or beneficial microbial processes. Therefore, these targets should be prioritized only when pathogen selectivity, crop safety, microbiome compatibility and delivery feasibility can be demonstrated, and they are more appropriate as sensitization or combination targets than as stand-alone antibacterial strategies.

Taken together, these considerations indicate that anti-virulence and anti-adaptation strategies should be developed primarily as components of integrated plant protection rather than as stand-alone replacements for existing products. In practical use, such compounds may be combined with copper bactericides, permitted antibiotics, biological control agents, induced-resistance agents, resistant cultivars or vector-management measures. These combinations may suppress virulence expression, colonization, biofilm formation or stress tolerance while improving the durability and reducing the input pressure of existing disease-control tools.

## 5. Technical Routes for Target Discovery and Validation

Target discovery in plant-pathogenic bacteria should follow an integrated workflow that links candidate identification with biological relevance and translational feasibility (Table 1). Genome annotation provided the initial functional framework for target nomination [66], whereas transcriptomic, proteomic and interactomic analyses help determine whether candidate genes or proteins were active during infection. Comparative genomics can further assess target conservation across pathogen species, strains or pathovars, whereas homology modeling and structural prediction can help identify potential ligand-binding pockets and druggable regions. In parallel, prediction of signal peptides, transmembrane domains, β-barrel structures and surface-exposed regions can inform cellular localization and target accessibility [38,42,60]. Functional validation can be supported by gene editing, gene silencing, heterologous expression, chemical inhibition and plant infection assays. For uncultured or fastidious pathogens, culturable relatives, heterologous systems, low-input transcriptomics and transient plant-expression assays are particularly useful for bridging annotation-based predictions with experimental validation.

Across the target classes discussed below, the level of evidence varies substantially, ranging from plant-pathogen or plant-assay validation to conceptual transfer from medical or model-bacterial systems. Therefore, each target class should be interpreted in relation to experimental evidence, plant-system validation, delivery feasibility, resistance risk and translational maturity, rather than molecular druggability alone. Target validation should not rely solely on inhibition zones or MIC values, because antibacterial activity in simplified culture assays does not necessarily predict disease-control performance *in planta* [31]. Candidate targets must be expressed in relevant infection niches, and the corresponding agents must reach the appropriate plant tissues, bacterial compartments and disease stages. For foliar pathogens, evaluation should consider ultraviolet exposure, rainfall wash-off and leaf-surface waxes [27]. For xylem- or phloem-associated pathogens, systemic movement, vascular delivery and plant metabolism are critical constraints [11,16,71]. In vector-borne diseases, target assessment should also include pathogen acquisition, persistence and transmission within insect vectors [128,129]. Robust target prioritization should therefore integrate infection-stage expression, conservation, pathogen selectivity, host and microbiome safety, delivery feasibility, resistance risk, field stability and translational feasibility. Candidate targets should also be evaluated in relation to scalability, production cost, regulatory acceptability, grower adoption and compatibility with integrated disease management, because biologically promising targets may not necessarily lead to socio-economically feasible disease-control products.

## 6. Potential Control Targets in CLas

### 6.1. Special Features of HLB and Implications for CLas Target Prioritization

Citrus Huanglongbing is one of the most destructive citrus diseases worldwide and is primarily associated with the phloem-limited α-proteobacterium “*Candidatus* Liberibacter asiaticus” (CLas) [13]. CLas is an insect-transmitted bacterium that cycles between citrus phloem and the Asian citrus psyllid, and its stable maintenance in artificial culture remains difficult [14,15,16,71,128]. It has a reduced genome, limited metabolic capacity, uneven distribution in planta, low bacterial abundance and delayed symptom development [15,16,71,91,134,135,136,137,138]. Together, these biological features make compound evaluation, target validation and field translation particularly challenging for HLB control [16,71].

Unlike typical T3SS-dependent plant pathogens, such as *Xanthomonas*, *P. syringae* and *Ralstonia* [46,47,48], CLas target prioritization cannot simply follow the conventional Hrp/T3SS-centered framework. Instead, CLas encodes multiple Sec-dependent secreted proteins and candidate effectors, some of which interfere with host immunity, protein degradation, vesicle trafficking or organelle function [60,61,62,64,134]. Therefore, target prioritization in CLas should integrate infection-stage expression with target accessibility and functional relevance to key features of CLas biology, including phloem restriction, Sec-dependent secretion and host-dependent metabolic adaptation [16,60,91].

### 6.2. Data Integration and Prioritization Criteria

**Figure 2:**
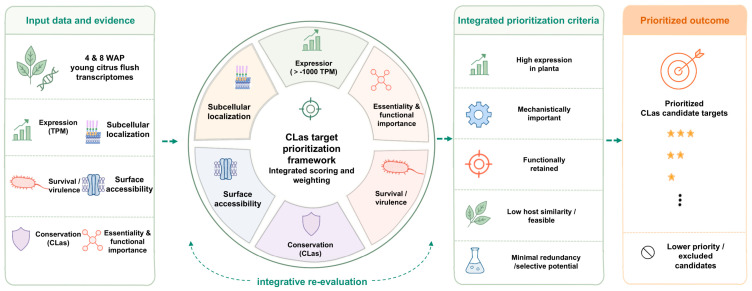
Framework-guided prioritization workflow for candidate control targets in *Candidatus* Liberibacter asiaticus (CLas). Candidate target nomination was performed by integrating CLas A4 genome annotation (CP010804.2) with young-flush transcript-abundance data from two infection-stage time points, 4 and 8 weeks after pathogen exposure. Mean TPM, maximum TPM and the 8 WAP/4 WAP ratio were used as expression-support indicators, but no single TPM value was used as an absolute inclusion threshold. Genes with strong expression were prioritized only when supported by biological function, whereas moderately expressed genes were retained when they belonged to mechanistically important target classes, such as Sec-dependent export, envelope/Bam assembly, nutrient transport, redox adaptation, core DNA/RNA processes or cell division. Candidate ranking further considered predicted localization or accessibility, relevance to survival, colonization, virulence or stress adaptation, conservation potential, validation feasibility, delivery feasibility and translational constraints. Genes were deprioritized when they were pseudogenes, poorly annotated, weakly expressed without clear mechanistic relevance, or lacked apparent accessibility or validation feasibility. The resulting High-, Medium-, Low- and Unknown-priority categories represent preliminary, hypothesis-generating nominations for downstream validation rather than direct evidence of essentiality, conservation, druggability, delivery feasibility or field applicability.

To prioritize potential control targets in CLas, we integrated CLas strain A4 genome (CP010804.2) annotation with genome-wide CLas transcript-abundance data (SRA: PRJNA1176309) from infected young citrus flushes [135,139]. The annotation dataset included CDS, tRNA, rRNA, ncRNA and tRNA features, together with locus tags, genomic coordinates, strand information, predicted products, EC numbers, protein IDs, pseudogene status, notes and inference evidence. The transcript-abundance dataset provided TPM (transcripts per kilobase million) values at 4 and 8 WAP (weeks after pruning). After matching records by locus tag, mean TPM, maximum TPM, the 8 WAP/4 WAP expression ratio and expression categories were calculated. Target nomination was performed as a qualitative, framework-guided ranking rather than a strict numerical scoring procedure. Mean TPM, maximum TPM and the 8 WAP/4 WAP ratio were used as expression evidence, but candidate priority was assigned only when transcript support converged with mechanistic target class, functional relevance, predicted accessibility and validation feasibility, as summarized in Figure 2.

The integrated dataset contained 1123 entries, including 1067 CDS records and 56 non-coding RNA records. Twenty-seven of these entries were annotated as pseudogenes. Functional classification showed that hypothetical or DUF-containing proteins, transporters, protein-synthesis genes, DNA/RNA core-process genes, energy-metabolism genes, redox and stress-adaptation genes, and protein secretion/export genes represented major components of the CLas A4 annotation. This pattern is consistent with genome reduction and the high proportion of functionally uncharacterized proteins in CLas [15,16,71,98].

Candidate targets were therefore prioritized using a qualitative, framework-guided approach derived from the general criteria summarized in Table 1. As summarized in Figure 2, candidate CLas targets were prioritized using a qualitative, framework-guided workflow derived from the general target-classification principles discussed in Section 3 and Section 4 and the prioritization criteria summarized in Table 1. In this workflow, genome annotation and infection-stage transcript abundance were treated as input evidence rather than as sufficient criteria for target selection. Transcript abundance was used to indicate whether a gene was active in young citrus flushes at biologically relevant infection stages, but it was not considered an independent indicator of target value. Instead, each candidate was evaluated through six linked layers: (i) infection-stage expression in young citrus flushes; (ii) assignment to one of the mechanistic target classes reviewed above, including envelope biogenesis, secretion/export, transport dependence, proteostasis, redox adaptation, nucleic-acid processes and cell division; (iii) inferred contribution to bacterial survival, colonization, virulence or stress adaptation; (iv) predicted cellular localization and potential accessibility, especially for surface-exposed, membrane-associated or secreted proteins; (v) feasibility of downstream validation through conservation analysis, heterologous expression, structural modeling, biochemical assays, surface-exposure tests or plant/vector-stage assays; and (vi) translational constraints, including delivery feasibility, selectivity, resistance risk and compatibility with integrated HLB management.

This Figure 2-based workflow explains why target priority was not determined by the expression level alone. Highly expressed genes were not automatically classified as high-priority targets if their functional relevance, accessibility or validation feasibility was unclear. Conversely, some moderately expressed genes, such as *bamA*, *secA*, *gyrB* and *ftsZ*, were retained because they mapped to mechanistically important and experimentally tractable target classes within the general framework. The final High-, Medium-, Low- and Unknown-priority categories therefore represent the integrated outcome of transcript evidence, mechanistic target class, functional relevance, predicted accessibility and validation feasibility, rather than a simple expression-ranking result. Accordingly, the 45 core CLas candidates should be interpreted as a framework-guided, hypothesis-generating target set, not as a list of experimentally validated antibacterial targets. Future work should refine this list through conservation screening across representative CLas isolates, structural and druggability assessment, heterologous functional validation, surface-exposure assays, compound or peptide screening, and citrus or psyllid-stage delivery experiments.

Accordingly, High-, Medium-, Low- and Unknown-priority categories were assigned on the basis of combined evidence rather than expression alone. For qualitative ranking, High-priority candidates were defined as genes supported by multiple convergent criteria, typically including infection-stage expression, assignment to a mechanistically important target class, inferred relevance to survival, virulence or stress adaptation, and predicted accessibility or feasible downstream validation. Medium-priority candidates met some but not all of these criteria, such as clear biological relevance but weaker support from expression, accessibility or validation feasibility. Low-priority candidates had limited translational value under the available evidence, whereas Unknown candidates were retained when annotation or functional interpretation was insufficient. This ranking was therefore used to reduce subjectivity in preliminary target nomination, but it should not be interpreted as quantitative evidence of essentiality, druggability or field applicability.

Using this framework, 45 core potential targets were selected from the High- and Medium-priority candidate pool by jointly considering TPM expression, biological relevance to CLas, theoretical accessibility, category balance and validation feasibility (Table 2). This list does not imply confirmed essentiality or proven chemical vulnerability, but instead provides a structured candidate set for downstream conservation analysis, structural prediction, heterologous expression, surface-exposure validation, small-molecule screening and plant- or vector-stage assays.

### 6.3. Core Target Classes and Expression Features

**Figure 3:**
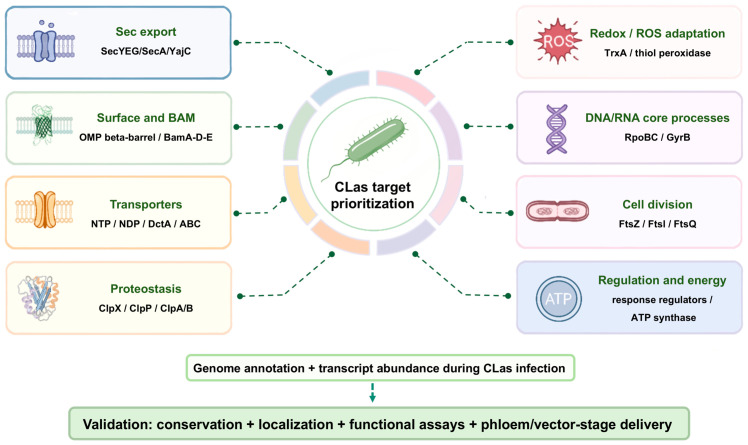
Hypothesis-generating candidate control-target classes in “*Candidatus* Liberibacter asiaticus” inferred from genome annotation and young-flush transcript abundance. The schematic highlights Sec-dependent export, outer-membrane and Bam-associated proteins, nutrient transporters, Clp-mediated proteostasis, thioredoxin-linked redox adaptation, RNA polymerase, DNA topoisomerase and cell-division proteins. These classes are prioritized candidate groups rather than functionally validated targets, and the grouping indicates whether a class is mainly surface-accessible, growth-essential, anti-virulence/adaptation-related or supported by high transcript abundance. Functional importance, essentiality, druggability, delivery feasibility and field relevance require downstream validation.

Mean TPM values in Table 2 were used only as descriptive indicators of transcript abundance and were not treated as direct evidence of biological importance, essentiality or druggability. Target prioritization also considered expression consistency between 4 and 8 WAP, mechanistic target class, inferred functional relevance, predicted localization or accessibility, potential host similarity, conservation potential and validation feasibility. The 45 core targets cover nine functional classes, including protein secretion and effector export, outer-membrane/surface structure and colonization, transporters/nutrient acquisition/drug delivery, cell division/cell wall, protein quality control/stress adaptation, redox/metal/ROS stress, core DNA/RNA processes, regulatory/signaling systems, and energy metabolism/respiratory chain (Table 2, Figure 3). Outer-membrane/surface and transporter targets are notable for their high expression and potential accessibility (Table 2). Sec-system targets are consistent with the established importance of CLas Sec-dependent effectors [60,61,62,63,64]. Clp-system and ROS-adaptation targets are consistent with the need to maintain protein homeostasis and redox balance in the phloem environment [65,98]. Thus, the CLas target classes identified here should be regarded as preliminary target nominations rather than validated antibacterial targets. Their prioritization is based mainly on genome annotation, infection-stage transcript abundance and framework-guided biological interpretation. Before practical development, these candidates require functional validation, conservation screening across representative CLas isolates, confirmation of expression or activity in citrus and psyllid-associated stages, assessment of surface accessibility or intracellular reachability, structural and druggability analysis, and evaluation of delivery feasibility in phloem- or vector-stage systems.

### 6.4. Main Prioritized CLas Target Groups and Validation Routes

Representative high-priority CLas candidates were selected according to the qualitative prioritization framework described in Figure 2. Candidate inclusion was supported by relatively high transcript abundance at one or both infection-stage time points, assignment to mechanistically important target classes, inferred relevance to survival, secretion, nutrient acquisition, proteostasis, redox adaptation or cell division, and predicted membrane association, surface exposure, secretion or experimental tractability (Table 3). These criteria were used to nominate candidates for downstream validation and should not be interpreted as quantitative evidence of essentiality, druggability or field applicability (Table 3).

The Sec-dependent secretion pathway may represent a potential anti-virulence target axis among the prioritized CLas candidates, but this possibility requires further functional, accessibility and delivery validation. The core candidate list included secY (CD16_00550), secG (CD16_00350), yajC (CD16_04980), secA (CD16_00990) and a protein annotated as a type II secretion system F family protein (CD16_02395) (Table 3). In the canonical Sec system, SecYEG forms the protein-translocation channel, SecA provides the ATP-dependent motor activity and YajC functionally cooperates with the SecDF system during protein export [38,39,40,41,42]. Given that multiple CLas Sec-dependent secreted proteins and candidate effectors had been reported, the prioritization of Sec-system components suggested that this pathway might influence protein localization, membrane homeostasis and the deployment of host-interaction factors [60,61,62,63,64]. However, Sec-dependent export should be interpreted as a candidate target axis rather than an immediately deployable control target. Because the Sec system is a conserved and fundamental protein-translocation pathway, Sec-directed inhibition requires careful assessment of pathogen selectivity, phloem delivery, effects on beneficial microbiota, and citrus or psyllid safety. For CLas, such targets are therefore more suitable for mechanistic validation and delivery-benchmark studies before practical disease-control development.

Outer-membrane and surface-associated proteins constituted another major group of prioritized CLas candidates with potential accessibility. This group included outer-membrane β-barrel proteins (CD16_03010 and CD16_04790), the OmpA family protein (CD16_03255), and BamA/BamD/BamE assembly factors (CD16_02135, CD16_05235 and CD16_03475) (Table 3). Among them, CD16_03010 showed extremely high TPM values at both time points, suggesting that it may represent a prominent envelope-associated component during young-flush infection (Table 3). Because CLas is phloem-limited and cannot be routinely maintained in stable culture, the accessibility of these outer-membrane or surface-associated proteins remains predicted rather than experimentally confirmed. These candidates should therefore be prioritized for topology prediction, β-barrel modeling, heterologous membrane-expression assays, surface-exposure validation, antibody- or peptide-binding tests, and confirmation of accessibility in citrus phloem or psyllid-associated stages before they are considered practical recognition or delivery targets.

Transporter candidates were prioritized as potential vulnerabilities associated with the host-dependent lifestyle of CLas. The core candidate set included the NTP/NDP exchange transporter (CD16_00970), the substrate-binding domain-containing transporter (CD16_00200), the cation:dicarboxylate symporter (CD16_05220), EamA family transporters (CD16_03120 and CD16_00840), the TSUP family transporter (CD16_04380), DctA (CD16_01285) and the phosphate ABC transporter ATP-binding protein (CD16_02485) (Table 3). These proteins may participate in the uptake or exchange of nucleotides, organic acids, amino acids, metal ions, phosphate or cofactor-related substrates, consistent with the reduced genome and host-dependent metabolic features of CLas. Accordingly, these transporters should be regarded as putative transporter-associated vulnerabilities rather than validated nutrient-deprivation targets or delivery-entry routes. Their functional relevance requires substrate prediction, heterologous expression, uptake assays, inhibitor-sensitivity tests and confirmation of expression or activity in citrus phloem or psyllid-associated stages.

Clp-mediated proteostasis and redox homeostasis also emerged as prominent candidate vulnerability classes (Table 2 and Table 3). ClpA, ClpX, ClpP, ClpB and ClpS may help maintain proteome stability under citrus phloem and psyllid-associated stress, whereas thioredoxin-disulfide reductase, thioredoxin, thiol peroxidase and related redox proteins may contribute to ROS detoxification and thiol-disulfide regulation [65,88,124,125,126]. These systems may be suitable for exploratory combination studies with induced-resistance agents, ROS-enhancing treatments, copper sensitization or other stress-amplifying approaches. Core-process targets, including RNA polymerase subunits, DNA gyrase, topoisomerase-related proteins and FtsZ/FtsI-associated cell-division proteins, were retained because they have high mechanistic value for structural modeling, heterologous validation and benchmark inhibition assays.

In CLas, conserved systems such as Sec-dependent export, Clp-mediated proteostasis, redox adaptation, RNA polymerase, DNA topoisomerases and cell-division proteins should currently be regarded mainly as mechanistic validation targets, research tools or delivery-system benchmarks rather than immediately deployable field-control targets. Their practical value depends on pathogen selectivity, phloem or vector-stage delivery, crop safety, microbiome compatibility and field efficacy, all of which require downstream validation.

Overall, the candidate targets summarized in Table 3 and discussed above are currently supported mainly by genome annotation, infection-stage transcript abundance and inferred functional relevance, rather than by direct functional validation in CLas. Their practical use is limited by several shared bottlenecks, including the lack of a stable CLas culture system, uncertain accessibility in citrus phloem or psyllid-associated stages, delivery feasibility, pathogen selectivity, non-target effects and field applicability. Therefore, these candidates should be viewed as prioritized hypotheses for staged validation rather than immediately deployable control targets.

### 6.5. Limitations and Translation Pathways for CLas Targets

Candidate CLas targets require further validation before they can support disease-control applications. Initial prioritization should first confirm that candidate genes are conserved across representative CLas isolates, expressed in relevant citrus or psyllid-associated stages, and predicted to be accessible through membrane localization, secretion signals, β-barrel structures or surface-exposed regions. However, these features alone are not sufficient to establish target value. Functional validation is therefore the next critical step. A practical CLas validation pipeline should proceed from target nomination to staged experimental confirmation. Candidate targets should first be examined for conservation across representative CLas genomes, followed by confirmation of expression across citrus tissues and psyllid-associated stages. Predicted localization, membrane association, secretion signals or surface accessibility should then be evaluated by bioinformatic and heterologous-expression approaches. Subsequent validation should include surrogate-system testing, biochemical or interaction assays, assessment of chemical accessibility or delivery feasibility, citrus- or psyllid-stage assays, and evaluation of crop, vector and microbiome safety.

Delivery and efficacy validation represent the final translational bottleneck. Because CLas is phloem-limited and insect-transmitted, potential candidate targets must be evaluated using delivery strategies compatible with citrus phloem and/or psyllid-associated stages, such as trunk injection, root uptake, nanocarriers, peptide-based delivery, antimicrobial peptides, RNA or antisense molecules and vector-stage interventions [16,71,93,128,129]. Ultimately, target value should be judged not only by molecular essentiality or inhibitory activity, but also by whether the intervention can reach the relevant tissue, reduce CLas fitness or transmission, and remain compatible with citrus growth, field deployment and integrated HLB management.

## 7. Conclusions and Perspectives

Control-target research in plant-pathogenic bacteria is moving beyond single bactericidal endpoints toward integrated intervention at the pathogen–host–environment interface. Growth-essential targets remain indispensable because they provide clear biochemical mechanisms and measurable inhibition endpoints. Cell-wall synthesis, outer-membrane biogenesis, nucleic-acid metabolism, protein synthesis, energy metabolism, nutrient transport and cell division continue to form the mechanistic foundation for antibacterial target discovery. However, the ecological features of plant-pathogenic systems, including leaf-surface exposure, vascular localization, insect transmission, microbiome interactions and field-delivery constraints, limit direct extrapolation from medical antibiotic discovery.

Anti-virulence and anti-adaptation targets broaden this control landscape by focusing on infection processes rather than bacterial viability alone. T3SS, TAL effector–host susceptibility gene axes, quorum sensing, biofilms, extracellular polysaccharides, secretion systems, plant cell-wall-degrading enzymes, resistance/sensitization systems and host immune regulators provide intervention points that may attenuate pathogenicity without necessarily imposing strong lethality. The inhibitor examples summarized in this review indicate that T3SS inhibitors, QS antagonists, quorum-quenching enzymes, outer-membrane-targeting strategies, induced-resistance agents and host-side peptide approaches are beginning to connect molecular plant pathology with agrochemical discovery. Nevertheless, many promising leads remain at the in vitro, greenhouse or controlled-environment stage, and their field translation will require improved stability, delivery, selectivity and ecological safety.

Future research should advance in four directions. First, high-quality functional annotation and infection-stage multi-omics should be generated for more plant-pathogenic bacterial species, strains and infection contexts. Second, structure-guided chemical biology should be integrated with plant-pathogen assays so that molecular inhibition can be linked to disease outcomes. Third, delivery technologies should be developed according to niche-specific barriers, especially for vascular and insect-transmitted pathogens. Fourth, ecological safety and regulatory feasibility should be evaluated early, including residue behavior, environmental fate, medically relevant resistance risk, effects on non-target microbiota, pollinators, soil microbial communities and aquatic organisms, crop physiology, and compatibility with regulatory approval requirements. Through these advances, target-based strategies for bacterial plant disease control should move from candidate nomination toward experimentally validated, delivery-compatible and resistance-managed interventions. Future research should prioritize infection-stage multi-omics, structure-guided target discovery, plant-pathogen validation systems, niche-specific delivery technologies, resistance-risk assessment, and early evaluation of ecological safety, non-target effects and regulatory feasibility. Such efforts may support greener, more precise and more durable management of bacterial plant diseases.

Among the target classes discussed in this review, potentially useful directions for agricultural antibacterial development are likely to be those that combine biological importance with feasible delivery and reduced resistance pressure. Envelope-associated targets and outer-membrane sensitization systems are attractive because they may improve compound entry and can be combined with copper compounds, peptides, phages or nanocarriers. Secretion systems, quorum-sensing pathways, biofilm-associated processes and host-susceptibility axes provide anti-virulence routes that may reduce disease development without relying solely on direct bactericidal activity. For host-dependent or vascular pathogens such as CLas, nutrient transporters, Sec-dependent secretion, surface-accessible proteins and stress-adaptation modules may be more realistic near-term targets than broad-spectrum core-process inhibitors. Classical targets such as RNA polymerase, DNA topoisomerases, ribosomes and FtsZ remain mechanistically important, but their agricultural use should prioritize low-cross-resistance chemotypes, crop safety, environmental compatibility and delivery efficiency.

Overall, this review emphasizes that target prioritization in plant-pathogenic bacteria must be multidimensional. Mechanistic tractability, infection-stage relevance, delivery feasibility, ecological safety and resistance-management value should be evaluated together. For CLas, this limitation is especially important because the pathogen cannot be routinely maintained in stable artificial culture and resides in phloem and psyllid-associated stages. Therefore, CLas candidate targets proposed from genome annotation and transcript-abundance evidence should be regarded as preliminary nominations that require functional, conservation, accessibility, delivery and field validation before practical application.

## Figures and Tables

**Table 1:** Criteria for prioritizing control targets in plant-pathogenic bacteria.

Criterion	Rationale	Preferred Evidence
Essentiality	Prioritizes targets required for growth or survival	Knockout/CRISPRi, conditional depletion, chemical inhibition
Infection-stage expression	Ensures the target is active in the relevant niche	RNA-seq, RT-qPCR, proteomics, in situ assays
Conservation	Supports broad strain or species coverage	Comparative genomics across isolates
Pathogen selectivity	Reduces host and microbiome toxicity	Host homolog comparison, active-site divergence
Cellular accessibility	Improves likelihood of compound or carrier access	Signal peptide, transmembrane, beta-barrel and surface-loop prediction
Delivery feasibility	Connects molecular target with agricultural use	Foliar, vascular, trunk injection, root uptake or vector-stage delivery tests
Resistance risk	Assesses durability of intervention	Mutation rate, target copy number, bypass pathways, combination potential
Field stability and safety	Determines translational value	UV/rainfastness, residues, phytotoxicity, microbiome effects
Translational and socio-economic feasibility	Ensures that biologically promising targets can be converted into practical, affordable and deployable disease-control solutions	Production scalability, formulation cost, regulatory acceptance, grower adoption, compatibility with integrated disease management

**Table 2:** Functional summary of 45 core potential CLas control targets.

Category	Number	Mean TPM	Representative Locus_Tag(s)
Protein secretion and effector export	5	1108.5	CD16_00550, CD16_02395, CD16_00350, CD16_04980, CD16_00990
Outer-membrane/surface structure and colonization	6	6016.6	CD16_03010, CD16_03475, CD16_04790, CD16_05235, CD16_03255, CD16_02135
Transporters/nutrient acquisition/drug delivery	8	1995.8	CD16_00970, CD16_00200, CD16_05220, CD16_03120, CD16_04380, CD16_01285, CD16_02485, CD16_00840
Cell division/cell wall	5	564.8	CD16_04200, CD16_05245, CD16_03265, CD16_05255, CD16_02455
Protein quality control/stress adaptation	5	1655.3	CD16_00700, CD16_00705, CD16_03785, CD16_00170, CD16_00175
Redox, metal and ROS stress	4	1478.2	CD16_00410, CD16_01885, CD16_01740, CD16_00765
Core DNA/RNA processes	5	2528.6	CD16_04095, CD16_00530, CD16_00065, CD16_00070, CD16_01925
Regulation/signaling system	4	1629.2	CD16_03875, CD16_03870, CD16_01735, CD16_02500
Energy metabolism/respiratory chain	3	1428.5	CD16_04840, CD16_04660, CD16_02900

Note: Mean TPM is shown as a transcript-abundance descriptor rather than an independent prioritization score. Candidate ranking also considered expression consistency, functional annotation, mechanistic relevance, inferred essentiality or virulence/stress-adaptation role, predicted localization/accessibility, potential host similarity, conservation potential, druggability and validation feasibility.

**Table 3:** Representative high-priority CLas candidate target nominations and suggested validation routes.

Category	Locus_Tag/Gene	Functional Annotation	Mean TPM	Rationale	Suggested Validation
Outer-membrane/surface structure and colonization	CD16_03010	Outer membrane beta-barrel protein	27,094	High expression and potential surface accessibility	Surface exposure, beta-barrel/Bam modeling, binding assays
Outer-membrane/surface structure and colonization	CD16_03475/bamE	Outer membrane protein assembly factor BamE	4535	High expression and potential surface accessibility	Surface exposure, beta-barrel/Bam modeling, binding assays
Outer-membrane/surface structure and colonization	CD16_02135/bamA	Outer membrane protein assembly factor BamA	807	Moderate expression, high envelope relevance, and potential surface accessibility	Surface exposure, beta-barrel/Bam modeling, binding assays
Outer-membrane/surface structure and colonization	CD16_05235/bamD	Outer membrane protein assembly factor BamD	1275	High expression and potential surface accessibility	Surface exposure, beta-barrel/Bam modeling, binding assays
Protein secretion and effector export	CD16_00550/secY	Preprotein translocase subunit SecY	1158	Links protein export with CLas effector output	Signal peptide prediction, Sec inhibition, effector secretion assays
Protein secretion and effector export	CD16_00350/secG	Preprotein translocase subunit SecG	1216	Links protein export with CLas effector output	Signal peptide prediction, Sec inhibition, effector secretion assays
Protein secretion and effector export	CD16_04980/yajC	Preprotein translocase subunit YajC	1281	Links protein export with CLas effector output	Signal peptide prediction, Sec inhibition, effector secretion assays
Protein secretion and effector export	CD16_00990/secA	Preprotein translocase subunit SecA	591	Links protein export with CLas effector output	Signal peptide prediction, Sec inhibition, effector secretion assays
Transporters/nutrient acquisition/drug delivery	CD16_00970	NTP/NDP exchange transporter	4510	Potential nutrient-dependence or delivery-entry vulnerability	Substrate prediction, heterologous uptake, inhibitor sensitivity
Transporters/nutrient acquisition/drug delivery	CD16_00200	Transporter substrate-binding domain-containing protein	3300	Potential nutrient-dependence or delivery-entry vulnerability	Substrate prediction, heterologous uptake, inhibitor sensitivity
Transporters/nutrient acquisition/drug delivery	CD16_05220	Cation:dicarboxylate symporter family transporter	1558	Potential nutrient-dependence or delivery-entry vulnerability	Substrate prediction, heterologous uptake, inhibitor sensitivity
Transporters/nutrient acquisition/drug delivery	CD16_01285/dctA	C4-dicarboxylate transporter DctA	1383	Potential nutrient-dependence or delivery-entry vulnerability	Substrate prediction, heterologous uptake, inhibitor sensitivity
Protein quality control/stress adaptation	CD16_00700/clpX	ATP-dependent Clp protease ATP-binding subunit ClpX	2824	Proteostasis and stress-adaptation vulnerability	Clp modulation, stress assays, heterologous complementation
Protein quality control/stress adaptation	CD16_00705	ATP-dependent Clp protease proteolytic subunit	1815	Proteostasis and stress-adaptation vulnerability	Clp modulation, stress assays, heterologous complementation
Redox, metal and ROS stress	CD16_01885/trxA	Thioredoxin	2421	Potential ROS and phloem-stress sensitization target	ROS challenge, thiol-redox assays, combination treatments
Redox, metal and ROS stress	CD16_00410	Thioredoxin-dependent thiol peroxidase	2206	Potential ROS and phloem-stress sensitization target	ROS challenge, thiol-redox assays, combination treatments
Core DNA/RNA processes	CD16_00070/rpoB	DNA-directed RNA polymerase subunit beta	2190	Mechanistically clear core-process target	Structural modeling, low-cross-resistance compound screening
Core DNA/RNA processes	CD16_00065/rpoC	DNA-directed RNA polymerase subunit beta	1941	Mechanistically clear core-process target	Structural modeling, low-cross-resistance compound screening
Core DNA/RNA processes	CD16_01925/gyrB	DNA topoisomerase (ATP-hydrolyzing) subunit B	845	Mechanistically clear core-process target	Structural modeling, low-cross-resistance compound screening
Cell division/cell wall	CD16_05245/ftsZ	Cell division protein FtsZ	675	Conserved cell-division target with clear phenotype	FtsZ modeling, morphology assays, delivery feasibility

Note: These candidates were nominated from genome annotation and infection-stage transcript-abundance data and should not be interpreted as functionally validated CLas control targets. Their essentiality, conservation, host/vector-stage activity, accessibility, druggability, delivery feasibility, non-target safety and field relevance require downstream validation. Suggested validation should be interpreted as a staged pipeline, including conservation screening, host/vector-stage expression confirmation, localization or accessibility assessment, heterologous or surrogate-system testing, delivery-feasibility evaluation, citrus/psyllid assays and non-target safety assessment.

## Data Availability

The CLas A4 genome annotation and young-flush transcript-abundance data used for target prioritization are available from GenBank (CP010804.2) and SRA (PRJNA1176309), respectively.

## Abbreviations

The following abbreviations are used in this manuscript:

CLas	*Candidatus* Liberibacter asiaticus
HLB	Huanglongbing
TPM	transcripts per kilobase million
WAP	weeks after pruning

## References

[1] Mansfield J., Genin S., Magori S., Citovsky V., Sriariyanum M., Ronald P., Dow M., Verdier V., Beer S.V., Machado M.A. (2012). Top 10 plant pathogenic bacteria in molecular plant pathology. Mol. Plant Pathol..

[2] Ryan R.P., Vorhölter F.J., Potnis N., Jones J.B., Van Sluys M.A., Bogdanove A.J., Dow J.M. (2011). Pathogenomics of Xanthomonas, understanding bacterium-plant interactions. Nat. Rev. Microbiol..

[3] Xin X.-F., Kvitko B., He S.Y. (2018). *Pseudomonas syringae*, what it takes to be a pathogen. Nat. Rev. Microbiol..

[4] Genin S., Denny T.P. (2012). Pathogenomics of the *Ralstonia solanacearum* species complex. Annu. Rev. Phytopathol..

[5] Vrancken K., Holtappels M., Schoofs H., Deckers T., Valcke R. (2013). Pathogenicity and infection strategies of the fire blight pathogen *Erwinia amylovora* in Rosaceae, state of the art. Microbiology.

[6] Charkowski A.O. (2018). The changing face of bacterial soft-rot diseases. Annu. Rev. Phytopathol..

[7] Reverchon S., Nasser W. (2013). Dickeya ecology, environment sensing and regulation of virulence programme. Environ. Microbiol. Rep..

[8] Hugouvieux-Cotte-Pattat N., Condemine G., Nasser W., Reverchon S. (1996). Regulation of pectinolysis in *Erwinia chrysanthemi*. Annu. Rev. Microbiol..

[9] Pérombelon M.C.M. (2002). Potato diseases caused by soft rot erwinias, an overview of pathogenesis. Plant Pathol..

[10] Goodner B., Hinkle G., Gattung S., Miller N., Blanchard M., Qurollo B., Goldman B.S., Cao Y., Askenazi M., Halling C. (2001). Genome sequence of the plant pathogen and biotechnology agent Agrobacterium tumefaciens C58. Science.

[11] Chatterjee S., Almeida R.P.P., Lindow S. (2008). Living in two worlds, the plant and insect lifestyles of *Xylella fastidiosa*. Annu. Rev. Phytopathol..

[12] Almeida R.P.P., Nunney L. (2015). How do plant diseases caused by *Xylella fastidiosa* emerge?. Plant Dis..

[13] Bové J.M. (2006). Huanglongbing, a destructive, newly-emerging, century-old disease of citrus. J. Plant Pathol..

[14] Jagoueix S., Bové J.M., Garnier M. (1994). The phloem-limited bacterium of greening disease of citrus is a member of the alpha subdivision of the Proteobacteria. Int. J. Syst. Bacteriol..

[15] Zheng Z., Chen J., Deng X. (2018). Historical perspectives, management, and current research of citrus HLB in Guangdong Province of China, where the disease has been endemic for over a hundred years. Phytopathology.

[16] Blaustein R.A., Lorca G.L., Teplitski M. (2018). Challenges for managing *Candidatus* Liberibacter spp. (Huanglongbing disease pathogen), Current control measures and future directions. Phytopathology.

[17] McManus P.S., Stockwell V.O., Sundin G.W., Jones A.L. (2002). Antibiotic use in plant agriculture. Annu. Rev. Phytopathol..

[18] Lamichhane J.R., Dachbrodt-Saaydeh S., Kudsk P., Messéan A. (2016). Toward a reduced reliance on conventional pesticides in European agriculture. Plant Dis..

[19] Stockwell V.O., Duffy B. (2012). Use of antibiotics in plant agriculture. Rev. Sci. Tech..

[20] Verhaegen M., Bergot T., Liebana E., Stancanelli G., Streissl F., Mingeot-Leclercq M.P., Mahillon J., Bragard C. (2023). On the use of antibiotics to control plant pathogenic bacteria, a genetic and genomic perspective. Front. Microbiol..

[21] Cooksey D.A. (1990). Genetics of bactericide resistance in plant pathogenic bacteria. Annu. Rev. Phytopathol..

[22] Marco G.M., Stall R.E. (1983). Control of bacterial spot of pepper initiated by strains of *Xanthomonas campestris* pv. vesicatoria that differ in sensitivity to copper. Plant Dis..

[23] Voloudakis A.E., Reignier T.M., Cooksey D.A. (2005). Regulation of resistance to copper in *Xanthomonas axonopodis* pv. vesicatoria. Appl. Environ. Microbiol..

[24] Behlau F., Canteros B.I., Minsavage G.V., Jones J.B., Graham J.H. (2011). Molecular characterization of copper resistance genes from *Xanthomonas citri* subsp. citri and *Xanthomonas alfalfae* subsp. citrumelonis. Appl. Environ. Microbiol..

[25] Islam T., Haque M.A., Barai H.R., Istiaq A., Kim J.-J. (2024). Antibiotic resistance in plant pathogenic bacteria, recent data and environmental impact of unchecked use and the potential of biocontrol agents as an eco-friendly alternative. Plants.

[26] Sundin G.W., Wang N. (2018). Antibiotic resistance in plant-pathogenic bacteria. Annu. Rev. Phytopathol..

[27] Khan S.J., Osborn A.M., Eswara P.J. (2021). Effect of sunlight on the efficacy of commercial antibiotics used in agriculture. Front. Microbiol..

[28] Tang J., Tong X., Chen Y., Wu Y., Zheng Z., Kayitmazer A.B., Ahmad A., Ramzan N., Yang J., Huang Q. (2023). Deposition and water repelling of temperature-responsive nanopesticides on leaves. Nat. Commun..

[29] Vincent C.I., Hijaz F., Pierre M., Killiny N. (2022). Systemic uptake of oxytetracycline and streptomycin in huanglongbing-affected citrus groves after foliar application and trunk injection. Antibiotics.

[30] Legein M., Smets W., Vandenheuvel D., Eilers T., Muyshondt B., Prinsen E., Samson R., Lebeer S. (2020). Modes of action of microbial biocontrol in the phyllosphere. Front. Microbiol..

[31] Ossola R., Farmer D.K. (2024). The chemical landscape of leaf surfaces and its interaction with the atmosphere. Chem. Rev..

[32] Lamichhane J.R., Osdaghi E., Behlau F., Köhl J., Jones J.B., Aubertot J.N. (2018). Thirteen decades of antimicrobial copper compounds applied in agriculture. A review. Agron. Sustain. Dev..

[33] Rasko D.A., Sperandio V. (2010). Anti-virulence strategies to combat bacteria-mediated disease. Nat. Rev. Drug Discov..

[34] Clatworthy A.E., Pierson E., Hung D.T. (2007). Targeting virulence, a new paradigm for antimicrobial therapy. Nat. Chem. Biol..

[35] Baron C. (2010). Antivirulence drugs to target bacterial secretion systems. Curr. Opin. Microbiol..

[36] Cegelski L., Marshall G.R., Eldridge G.R., Hultgren S.J. (2008). The biology and future prospects of antivirulence therapies. Nat. Rev. Microbiol..

[37] Allen R.C., Popat R., Diggle S.P., Brown S.P. (2014). Targeting virulence, can we make evolution-proof drugs?. Nat. Rev. Microbiol..

[38] Theuretzbacher U., Blasco B., Duffey M., Piddock L.J. (2023). Unrealized targets in the discovery of antibiotics for Gram-negative bacterial infections. Nat. Rev. Drug Discov..

[39] Brown E.D., Wright G.D. (2016). Antibacterial drug discovery in the resistance era. Nature.

[40] Silver L.L. (2011). Challenges of antibacterial discovery. Clin. Microbiol. Rev..

[41] Lewis K. (2013). Platforms for antibiotic discovery. Nat. Rev. Drug Discov..

[42] Butler M.S., Vollmer W., Goodall E.C.A., Capon R.J., Henderson I.R., Blaskovich M.A. (2024). A review of antibacterial candidates with new modes of action. ACS Infect. Dis..

[43] Vollmer W., Blanot D., de Pedro M.A. (2008). Peptidoglycan structure and architecture. FEMS Microbiol. Rev..

[44] den Blaauwen T., Hamoen L.W., Levin P.A. (2017). The divisome at 25, the road ahead. Curr. Opin. Microbiol..

[45] Kusuma K.D., Payne M., Ung A.T., Bottomley A.L., Harry E.J. (2019). FtsZ as an antibacterial target, status and guidelines for progressing this avenue. ACS Infect. Dis..

[46] Büttner D., Bonas U. (2010). Regulation and secretion of Xanthomonas virulence factors. FEMS Microbiol. Rev..

[47] Büttner D. (2012). Protein export according to schedule, architecture, assembly, and regulation of type III secretion systems from plant- and animal-pathogenic bacteria. Microbiol. Mol. Biol. Rev..

[48] Deng W., Marshall N.C., Rowland J.L., McCoy J.M., Worrall L.J., Santos A.S., Strynadka N.C.J., Finlay B.B. (2017). Assembly, structure, function and regulation of type III secretion systems. Nat. Rev. Microbiol..

[49] Alfano J.R., Collmer A. (2004). Type III secretion system effector proteins, double agents in bacterial disease and plant defense. Annu. Rev. Phytopathol..

[50] Toruño T.Y., Stergiopoulos I., Coaker G. (2016). Plant-pathogen effectors, cellular probes interfering with plant defenses in spatial and temporal manners. Annu. Rev. Phytopathol..

[51] Von Bodman S.B., Bauer W.D., Coplin D.L. (2003). Quorum sensing in plant-pathogenic bacteria. Annu. Rev. Phytopathol..

[52] Flemming H.C., Wingender J. (2010). The biofilm matrix. Nat. Rev. Microbiol..

[53] Hall-Stoodley L., Costerton J.W., Stoodley P. (2004). Bacterial biofilms, from the natural environment to infectious diseases. Nat. Rev. Microbiol..

[54] Morris C.E., Monier J.M. (2003). The ecological significance of biofilm formation by plant-associated bacteria. Annu. Rev. Phytopathol..

[55] Hutin M., Pérez-Quintero A.L., Lopez C., Szurek B. (2015). MorTAL Kombat, the story of defense against TAL effectors through loss-of-susceptibility. Front. Plant Sci..

[56] Oliva R., Ji C., Atienza-Grande G., Huguet-Tapia J.C., Perez-Quintero A., Li T., Eom J.S., Li C., Nguyen H., Liu B. (2019). Broad-spectrum resistance to bacterial blight in rice using genome editing. Nat. Biotechnol..

[57] Hu Y., Zhang J., Jia H., Sosso D., Li T., Frommer W.B., Yang B., White F.F., Wang N., Jones J.B. (2014). Lateral organ boundaries 1 is a disease susceptibility gene for citrus bacterial canker disease. Proc. Natl. Acad. Sci. U.S.A..

[58] Jia H., Orbović V., Jones J.B., Wang N. (2016). Modification of the PthA4 effector binding elements in Type I CsLOB1 promoter using Cas9/sgRNA to produce transgenic Duncan grapefruit alleviating XccΔpthA4,dCsLOB1.3 infection. Plant Biotechnol. J..

[59] Baltenneck J., Reverchon S., Hommais F. (2021). Quorum sensing regulation in phytopathogenic bacteria. Microorganisms.

[60] Prasad S., Xu J., Zhang Y., Wang N. (2016). SEC-translocon dependent extracytoplasmic proteins of *Candidatus* Liberibacter asiaticus. Front. Microbiol..

[61] Zhang S., Wang X., He J., Zhang S., Zhao T., Fu S., Zhou C. (2023). A Sec-dependent effector, CLIBASIA_04425, contributes to virulence in *Candidatus* Liberibacter asiaticus. Front. Plant Sci..

[62] Huang G., Chang X., Hu Y., Li F., Wang N., Li R. (2024). SDE19, a SEC-dependent effector from *Candidatus* Liberibacter asiaticus, suppresses plant immunity and targets Citrus sinensis Sec12 to interfere with vesicle trafficking. PLoS Pathog..

[63] Hu Y., Lu N., Bao K., Liu S., Li R., Huang G. (2025). Swords and shields, the war between *Candidatus* Liberibacter asiaticus and citrus. Front. Plant Sci..

[64] Yang C., Ancona V. (2022). An overview of the mechanisms against *Candidatus* Liberibacter asiaticus, virulence targets, citrus defenses and microbiome. Front. Microbiol..

[65] Pitino M., Armstrong C.M., Duan Y. (2017). Molecular mechanisms behind the accumulation of ATP and H_2_O_2_ in citrus plants in response to *Candidatus* Liberibacter asiaticus infection. Hortic. Res..

[66] Pallen M.J., Wren B.W. (2007). Bacterial pathogenomics. Nature.

[67] Ranf S., Gisch N., Schäffer M., Illig T., Westphal L., Knirel Y.A., Sánchez-Carballo P.M., Zähringer U., Hückelhoven R., Lee J. (2015). A lectin S-domain receptor kinase mediates lipopolysaccharide sensing in *Arabidopsis thaliana*. Nat. Immunol..

[68] Ryan R.P., Dow J.M. (2011). Communication with a growing family, diffusible signal factor (DSF) signaling in bacteria. Trends Microbiol..

[69] Guilhabert M.R., Kirkpatrick B.C. (2005). Identification of *Xylella fastidiosa* antivirulence genes, hemagglutinin adhesins contribute to biofilm maturation and colonization and attenuate virulence. Mol. Plant Microbe Interact..

[70] Newman K.L., Almeida R.P.P., Purcell A.H., Lindow S.E. (2004). Cell-cell signaling controls *Xylella fastidiosa* interactions with both insects and plants. Proc. Natl. Acad. Sci. USA.

[71] Merfa M.V., Pérez-López E., Naranjo E., Jain M., Gabriel D.W., De La Fuente L. (2019). Progress and obstacles in culturing *Candidatus* Liberibacter asiaticus, the bacterium associated with huanglongbing. Phytopathology.

[72] Barb A.W., Zhou P. (2008). Mechanism and inhibition of LpxC, an essential zinc-dependent deacetylase of bacterial lipid A synthesis. Curr. Pharm. Biotechnol..

[73] Srinivas N., Jetter P., Ueberbacher B.J., Werneburg M., Zerbe K., Steinmann J., Van der Meijden B., Bernardini F., Lederer A., Dias R.L. (2010). Peptidomimetic antibiotics target outer-membrane biogenesis in Pseudomonas aeruginosa. Science.

[74] Kaur H., Jakob R.P., Marzinek J.K., Green R., Imai Y., Bolla J.R., Agustoni E., Robinson C.V., Bond P.J., Lewis K. (2021). The antibiotic darobactin mimics a β-strand to inhibit outer membrane insertase. Nature.

[75] Vaara M. (2019). Polymyxins and their potential next generation as therapeutic antibiotics. Front. Microbiol..

[76] Giovanardi D., Biondi E., Biondo N., Quiroga N., Modica F., Puopolo G., Pérez Fuentealba S. (2025). Sustainable and innovative biological control strategies against *Pseudomonas syringae* pv. tomato, *Pseudomonas savastanoi* pv. phaseolicola and *Xanthomonas* spp. affecting vegetable crops: A review. Front. Plant Sci..

[77] Buttimer C., McAuliffe O., Ross R.P., Hill C., O’Mahony J., Coffey A. (2017). Bacteriophages and bacterial plant diseases. Front. Microbiol..

[78] Tang Y., Zhou M., Yang C., Liu R., Du H., Ma M. (2024). Advances in isolated phages that affect *Ralstonia solanacearum* and their application in the biocontrol of bacterial wilt in plants. Lett. Appl. Microbiol..

[79] Peng A., Chen S., Lei T., Xu L., He Y., Wu L., Yao L., Zou X. (2017). Engineering canker-resistant plants through CRISPR/Cas9-targeted editing of the susceptibility gene CsLOB1 promoter in citrus. Plant Biotechnol. J..

[80] Huang C.-Y., Araujo K., Sánchez J.N., Kund G., Trumble J., Roper C., Godfrey K.E., Jin H. (2021). A stable antimicrobial peptide with dual functions of treating and preventing citrus Huanglongbing. Proc. Natl. Acad. Sci. USA.

[81] Hu J., Wang N. (2016). Evaluation of the spatiotemporal dynamics of oxytetracycline and its control effect against citrus Huanglongbing via trunk injection. Phytopathology.

[82] Dewdney M.M., Vashisth T., Diepenbrock L.M. (2025). 2025–2026 Florida Citrus Production Guide: Huanglongbing (Citrus Greening).

[83] Typas A., Banzhaf M., Gross C.A., Vollmer W. (2012). From the regulation of peptidoglycan synthesis to bacterial growth and morphology. Nat. Rev. Microbiol..

[84] Dajkovic A., Lutkenhaus J. (2006). Z ring as executor of bacterial cell division. J. Mol. Microbiol. Biotechnol..

[85] Kahan F.M., Kahan J.S., Cassidy P.J., Kropp H. (1974). The mechanism of action of fosfomycin (phosphonomycin). Ann. N. Y. Acad. Sci..

[86] Winn M., Goss R.J.M., Kimura K., Bugg T.D.H. (2010). Antimicrobial nucleoside antibiotics targeting cell wall assembly, Recent advances in structure–function studies and nucleoside biosynthesis. Nat. Prod. Rep..

[87] Sauvage E., Kerff F., Terrak M., Ayala J.A., Charlier P. (2008). The penicillin-binding proteins, structure and role in peptidoglycan biosynthesis. FEMS Microbiol. Rev..

[88] Collin F., Karkare S., Maxwell A. (2011). Exploiting bacterial DNA gyrase as a drug target, current state and perspectives. Appl. Microbiol. Biotechnol..

[89] Campbell E.A., Korzheva N., Mustaev A., Murakami K., Nair S., Goldfarb A., Darst S.A. (2001). Structural mechanism for rifampicin inhibition of bacterial RNA polymerase. Cell.

[90] Wilson D.N. (2014). Ribosome-targeting antibiotics and mechanisms of bacterial resistance. Nat. Rev. Microbiol..

[91] Jha G., Rajeshwari R., Sonti R.V. (2005). Bacterial type two secretion system secreted proteins, double-edged swords for plant pathogens. Mol. Plant Microbe Interact..

[92] Nivaskumar M., Francetic O. (2014). Type II secretion system, a magic beanstalk or a protein escalator. Biochim Biophys. Acta.

[93] Smith P.A., Romesberg F.E. (2012). Mechanism of action of the arylomycin antibiotics and effects of signal peptidase I inhibition. Antimicrob. Agents Chemother..

[94] Akula N., Zheng H., Han F.Q., Wang N. (2011). Discovery of novel SecA inhibitors of *Candidatus* Liberibacter asiaticus by structure-based design. Bioorg. Med. Chem. Lett..

[95] Brötz-Oesterhelt H., Beyer D., Kroll H.P., Endermann R., Ladel C., Schroeder W., Hinzen B., Raddatz S., Paulsen H., Henninger K. (2005). Dysregulation of bacterial proteolytic machinery by a new class of antibiotics. Nat. Med..

[96] Hurley K.A., Santos T.M.A., Nepomuceno G.M., Huynh V., Shaw J.T., Weibel D.B. (2016). Targeting the bacterial division protein FtsZ. J. Med. Chem..

[97] Bean G.J., Flickinger S.T., Westler W.M., McCully M.E., Sept D., Weibel D.B., Amann K.J. (2009). A22 disrupts the bacterial actin cytoskeleton by directly binding and inducing a low-affinity state in MreB. Biochemistry.

[98] Jain M., Munoz-Bodnar A., Gabriel D.W. (2017). Concomitant loss of the glyoxalase system and glycolysis makes the uncultured pathogen *Candidatus* Liberibacter asiaticus an energy scavenger. Appl. Environ. Microbiol..

[99] Fatima U., Senthil-Kumar M. (2015). Plant and pathogen nutrient acquisition strategies. Front. Plant Sci..

[100] Saxena D., Maitra R., Bormon R., Czekanska M., Meiers J., Titz A., Verma S., Chopra S. (2023). Tackling the outer membrane, facilitating compound entry into Gram-negative bacterial pathogens. npj Antimicrob. Resist..

[101] Deslandes L., Rivas S. (2012). Catch me if you can, bacterial effectors and plant targets. Trends Plant Sci..

[102] Macho A.P. (2016). Subversion of plant cellular functions by bacterial type-III effectors, beyond suppression of immunity. New Phytol..

[103] Yang F., Korban S.S., Pusey P.L., Elofsson M., Sundin G.W., Zhao Y. (2014). Small-molecule inhibitors suppress the expression of both type III secretion and amylovoran biosynthesis genes in *Erwinia amylovora*. Mol. Plant Pathol..

[104] Tao H., Fan S.S., Jiang S., Xiang X., Yan X., Zhang L.H., Cui Z.N. (2019). Small molecule inhibitors specifically targeting the type III secretion system of *Xanthomonas oryzae* on rice. Int. J. Mol. Sci..

[105] Fan S., Tian F., Fang L., Yang C.H., He C. (2019). Transcriptional responses of *Xanthomonas oryzae* pv. oryzae to type III secretion system inhibitor ortho-coumaric acid. BMC Microbiol..

[106] Tao H., Tian H., Jiang S., Xiang X., Lin Y., Ahmed W., Tang R., Cui Z.N. (2019). Synthesis and biological evaluation of 1,3,4-thiadiazole derivatives as type III secretion system inhibitors against *Xanthomonas oryzae*. Pestic. Biochem. Physiol..

[107] Yuan X., Yu M., Yang C.H. (2020). Innovation and application of the type III secretion system inhibitors in plant pathogenic bacteria. Microorganisms.

[108] Bogdanove A.J., Schornack S., Lahaye T. (2010). TAL effectors, finding plant genes for disease and defense. Curr. Opin. Plant Biol..

[109] Boch J., Bonas U. (2010). Xanthomonas AvrBs3 family-type III effectors, discovery and function. Annu. Rev. Phytopathol..

[110] Scholze H., Boch J. (2011). TAL effectors are remote controls for gene activation. Curr. Opin. Microbiol..

[111] Doyle E.L., Stoddard B.L., Voytas D.F., Bogdanove A.J. (2013). TAL effectors, highly adaptable phytobacterial virulence factors and readily engineered DNA-targeting proteins. Trends Cell Biol..

[112] Yang B., Sugio A., White F.F. (2006). Os8N3 is a host disease-susceptibility gene for bacterial blight of rice. Proc. Natl. Acad. Sci. USA.

[113] Streubel J., Pesce C., Hutin M., Koebnik R., Boch J., Szurek B. (2013). Five phylogenetically close rice SWEET genes confer TAL effector-mediated susceptibility to *Xanthomonas oryzae* pv. oryzae. New Phytol..

[114] Gordon J.E., Christie P.J. (2014). The Agrobacterium Ti plasmids. Microbiol. Spectr..

[115] Gelvin S.B. (2003). Agrobacterium-mediated plant transformation, the biology behind the gene-jockeying tool. Microbiol. Mol. Biol. Rev..

[116] Louws F.J., Wilson M., Campbell H.L., Cuppels D.A., Jones J.B., Shoemaker P.B., Sahin F., Miller S.A. (2001). Field control of bacterial spot and bacterial speck of tomato using a plant activator. Plant Dis..

[117] Walters D.R., Ratsep J., Havis N.D. (2013). Controlling crop diseases using induced resistance, challenges for the future. J. Exp. Bot..

[118] Zhao P., Sun Y., Chen X., Zhang J., Yang H., Hao X., Fang R., Ye J. (2025). A small peptide APP3-14 disrupts pathogen-insect mutualism by modulating plant MYC2-mediated olfactory defense. Plant Commun..

[119] Zhao P., Yang H., Sun Y., Zhang J., Gao K., Wu J., Zhu C., Yin C., Chen X., Liu Q. (2025). Targeted MYC2 stabilization confers citrus Huanglongbing resistance. Science.

[120] Miller M.B., Bassler B.L. (2001). Quorum sensing in bacteria. Annu. Rev. Microbiol..

[121] Fuqua C., Greenberg E.P. (2002). Listening in on bacteria, acyl-homoserine lactone signalling. Nat. Rev. Mol. Cell Biol..

[122] Whitehead N.A., Barnard A.M.L., Slater H., Simpson N.J.L., Salmond G.P.C. (2001). Quorum-sensing in Gram-negative bacteria. FEMS Microbiol. Rev..

[123] Flavier A.B., Clough S.J., Schell M.A., Denny T.P. (1997). Identification of 3-hydroxypalmitic acid methyl ester as a novel autoregulator controlling virulence in *Ralstonia solanacearum*. Mol. Microbiol..

[124] Jiang Q., Chen J., Yang C., Yin Y., Yao K. (2019). Quorum sensing, a prospective therapeutic target for bacterial diseases. BioMed Res. Int..

[125] Dong Y.H., Xu J.L., Li X.Z., Zhang L.H. (2000). AiiA, an enzyme that inactivates the acylhomoserine lactone quorum-sensing signal and attenuates Erwinia carotovora infection. Proc. Natl. Acad. Sci. USA.

[126] Yoshihara A., Shimatani M., Sakata M., Takemura C., Senuma W., Hikichi Y., Kai K. (2020). Quorum sensing inhibition attenuates the virulence of the plant pathogen *Ralstonia solanacearum* species complex. ACS Chem. Biol..

[127] Ryan R.P., An S.Q., Allan J.H., McCarthy Y., Dow J.M. (2015). The DSF family of cell-cell signals, an expanding class of bacterial virulence regulators. PLoS Pathog..

[128] Pelz-Stelinski K.S., Brlansky R.H., Ebert T.A., Rogers M.E. (2010). Transmission parameters for *Candidatus* Liberibacter asiaticus by Asian citrus psyllid (Hemiptera, Psyllidae). J. Econ. Entomol..

[129] Grafton-Cardwell E.E., Stelinski L.L., Stansly P.A. (2013). Biology and management of Asian citrus psyllid, vector of the huanglongbing pathogens. Annu. Rev. Entomol..

[130] Kaplan J.B. (2010). Biofilm dispersal, mechanisms, clinical implications, and potential therapeutic uses. J. Dent. Res..

[131] Harbut M.B., Vilchèze C., Luo X., Hensler M.E., Guo H., Yang B., Chatterjee A.K., Nizet V., Jacobs W.R., Schultz P.G. (2015). Auranofin exerts broad-spectrum bactericidal activities by targeting thiol-redox homeostasis. Proc. Natl. Acad. Sci. USA.

[132] Singh A., Kumar N., Tomar P.P.S., Bhose S., Ghosh D.K., Roy P., Sharma A.K. (2017). Characterization of a bacterioferritin comigratory protein family 1-Cys peroxiredoxin from *Candidatus* Liberibacter asiaticus. Protoplasma.

[133] Jain M., Munoz-Bodnar A., Gabriel D.W. (2019). *Candidatus* Liberibacter asiaticus peroxiredoxin suppresses oxylipin-mediated defense signaling in citrus. J. Plant Physiol..

[134] Clark K., Franco J.Y., Schwizer S., Pang Z., Hawara E., Liebrand T.W.H., Pagliaccia D., Zeng L., Gurung F.B., Wang P. (2018). An effector from the huanglongbing-associated pathogen targets citrus proteases. Nat. Commun..

[135] Zheng Z., Deng X., Chen J. (2014). Whole-genome sequence of “*Candidatus* Liberibacter asiaticus” from Guangdong, China. Genome Announc..

[136] Zheng Y., Li J., Zheng M., Li Y., Deng X., Zheng Z. (2024). Whole genome sequences of 135 “*Candidatus* Liberibacter asiaticus” strains from China. Sci. Data.

[137] Zheng Z., Bao M., Wu F., Van Horn C., Chen J., Deng X. (2018). A type 3 prophage of ‘*Candidatus* Liberibacter asiaticus’ carrying a restriction-modification system. Phytopathology.

[138] Zheng Z., Wu F., Kumagai L.B., Polek M., Deng X., Chen J. (2017). Two ‘*Candidatus* Liberibacter asiaticus’ strains recently found in California harbor different prophages. Phytopathology.

[139] Li J., Chen J., Huang W., Chen X., Li C., Xu M., Deng X., Zheng Z. (2025). Anatomical and dual transcriptomic analysis reveals the interaction of “*Candidatus* Liberibacter asiaticus” and citrus host in new shoots at different growth stage. Hortic. Plant J..

